# Heterogeneity of frequencies of motor neuron disease across ethnicities and geographical areas: focus on Arabic countries in the Mediterranean area

**DOI:** 10.1097/WCO.0000000000001415

**Published:** 2025-08-22

**Authors:** Giancarlo Logroscino, Stefano Giannoni-Luza, Daniele Urso, Nabila Hamdi

**Affiliations:** a Center for Neurodegenerative Diseases and the Aging Brain, Department of Clinical Research in Neurology, University of Bari ’Aldo Moro’, “Pia Fondazione Cardinale G. Panico”, Tricase, Lecce; b University of Bari Aldo Moro, Department of Translational Biomedicine and Neuroscience (DiBraiN), Bari, Apulia, Italy; c Department of Pharmacology and Toxicology, Faculty of Pharmacy and Biotechnology, The German University in Cairo, Cairo, Egypt

**Keywords:** amyotrophic lateral sclerosis, Arabic countries, Egypt, epidemiology, Global Burden of Disease, North Africa

## Abstract

**Purpose of review:**

Although amyotrophic lateral sclerosis (ALS) epidemiology has been increasingly characterized in many regions, data from Arabic countries remain limited. This review aims to summarize the current knowledge on the burden of ALS in Arabic Mediterranean countries, with a particular focus on Egypt.

**Recent findings:**

ALS exhibits significant geographic and ethnic variability in terms of incidence, phenotype, and genetic background. Data from the Global Burden of Disease Study 2021 show that Egypt has one of the lowest age-standardized rates of ALS incidence, prevalence, and mortality in the Mediterranean basin. During the past three decades, Egypt has seen a notable decline in ALS-related Disability-Adjusted Life Years and deaths, in contrast to neighboring countries. A national registry has recently been initiated to enhance epidemiological surveillance in the country.

**Summary:**

ALS in Arabic Mediterranean countries presents a distinct epidemiological profile. These differences likely reflect a combination of genetic, demographic, and healthcare-related factors. Strengthening national registries and promoting regional collaborations will be crucial for gaining a deeper understanding of the determinants of ALS in these underrepresented populations.

## INTRODUCTION

Amyotrophic laterals sclerosis (ALS) is a rare and rapidly progressive neurodegenerative disease [1]. Its rarity and aggressiveness make the study of ALS epidemiology particularly challenging [2]. According to EU definition, a disease is considered rare when its prevalence is under 50 per 100 000 individuals. Both incidence and prevalence of ALS are relatively low, but exhibit significant variability, with higher rates in Europe, North America, Australia, and New Zealand, and lower rates in Asia, Africa, and South America [3]. Incidence ranges from 1 to 4 per 100 000 person-years, while prevalence from 3 to 12 per 100 000 [4]. The origin of this difference remains unclear. Evidence suggests that ALS risk varies across continents and ethnicities, with some studies highlighting the potential role of ethnicity and underlying genetic differences [5]. The EURALS, a collaborative population-based study conducted in Europe, found an average incidence of 2.2 per 100 000 person-years for the general population [6]. One potential driver of incidence in Europe could be the distribution of the *C9ORF72* gene [7], which is associated with a more severe phenotype and the presence of behavioral disorders [8]. *C9ORF72* shows the highest prevalence in populations of Scandinavian origin [7].

The Global Burden of Disease (GBD) is a well-established project that, over the last 35 years, has aimed to describe the burden of 371 diseases and injuries across 21 regions, 204 countries, and more than 800 subregions worldwide, using a standardized protocol [9^▪▪^]. GBD is a valuable tool for exploring the variability of motor neuron disease (MND) [5]. It has gathered data from over one million sources as of 2021 to estimate the burden of diseases and injuries [9^▪▪^]. Two major demographic changes are driving the increasing burden of MND, which is projected to reach 500 000 new cases annually worldwide in the coming decades: increased life expectancy and population growth [10].

The burden of MND is expected to shift toward Asia, Latin America, and Africa in the next few decades due to the rapid increase in life expectancy in countries with significant demographic growth [10]. One area of particular interest for understanding the epidemiology of ALS is the Mediterranean region. In this region, the northern part (including Italy, Greece, and Spain) has a low frequency of ALS compared to other European countries, while the southern coast, which represents the northern part of Africa, also shows relatively low prevalence [3]. The Mediterranean coast of Africa is predominantly inhabited by Arabic populations. This area has a high prevalence and incidence of other neurodegenerative diseases, such as dementias, similar to patterns observed in Middle Eastern countries [11]. We have initiated a national registry in Egypt [12^▪▪^] with the goal of establishing a new data source and providing additional evidence on ALS epidemiology in an Arabic-speaking country.

In this paper we identify key characteristics of epidemiology of ALS in Arabic countries with a particular focus on Egypt, and compare these countries with Southern Europe and Africa within the framework of the GBD. 

**Box 1:**
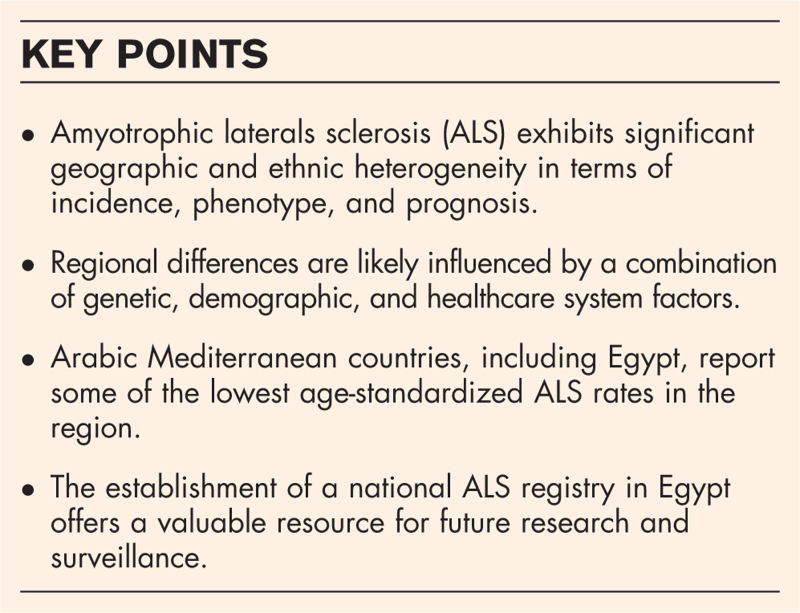
no caption available

## HETEROGENEITY OF CLINICAL PRESENTATION ACROSS THE WORLD

ALS exhibits marked phenotypic heterogeneity worldwide, with variations in clinical presentation, disease course, and associated features observed across diverse geographic regions and ancestral populations [1]. This variability, increasingly documented through population-based epidemiological studies [3,13], has significant implications not only for diagnosis and prognosis, but also for advancing our understanding of the disease's etiology [5].

A comprehensive meta-analysis [13] encompassing over 12 000 ALS cases from 40 geographic areas identified subcontinental origin as a key determinant of clinical heterogeneity, influencing factors such as sex distribution, age at onset, site of symptom onset, diagnostic latency, survival, and the frequency of familial ALS (fALS). For instance, the proportion of bulbar-onset cases is markedly higher in Northern Europe (approximately 45%) compared to regions such as East and West Asia, where the frequency is below 30% [13]. Similarly, the male-to-female ratio remains relatively stable in Europe (1.2–1.3), but increases significantly in regions such as West Asia, where it exceeds 1.7. These disparities may reflect underlying genetic susceptibility, as well as region-specific differences in healthcare access, sociocultural dynamics, and case reporting practices.

Age at symptom onset similarly varies in a geographically structured manner. While the mean age at onset in Europe and New Zealand hovers around 63–65  years, lower averages are reported in East Asia, the Middle East, and North Africa, where onset frequently occurs before age 60. Such variation may be partly attributable to demographic differences, such as population life expectancy, but also suggests regional differences in genetic architecture or environmental exposures [13].

Diagnostic delay, an important clinical metric, further highlights geographic disparities. In Europe and Oceania, the average delay is approximately 10–12  months, whereas in some North African countries (e.g., Libya), it can exceed 40  months. Similarly, median survival from symptom onset varies considerably, ranging from 24 to 25 months in Northern Europe to over 40  months in Central Asia and Iran suggesting both biological variability and disparities in health system performance [13].

Importantly, the heterogeneity of ALS is not only evident across ancestral groups but also within mixed populations [14–20]. A striking example comes from a population-based mortality study conducted in Cuba, a country with a highly heterogeneous ethnic composition (65% mixed, 25% white, and 10% Black) and universal access to high-standard healthcare. In this setting, ALS mortality was found to be lowest among individuals of mixed ancestry (0.55 per 100 000 person-years), compared to 0.93 among white individuals and 0.87 among Black individuals, despite comparable healthcare access and diagnostic pathways [19]. These findings support the idea that ancestry has a biological influence on ALS risk, independent of geographic factors, likely mediated by genetic background.

The genetic underpinnings of ALS heterogeneity have received increasing attention. The *C9orf72* hexanucleotide repeat expansion, the most common genetic cause of ALS and frontotemporal dementia (FTD) in populations of Northern European descent [7,21], is also associated with a broader neuropsychiatric profile, including cognitive impairment and psychosis, and movement disorders such as parkinsonism and chorea [22]. However, this mutation is very low in many Asian populations [23,24,25]. In contrast, SOD1 mutations are more prevalent in Asian populations, particularly in familial ALS, whereas their frequency remains low in sporadic cases, especially in Caucasian populations [26^▪^]. Founder mutations in *SOD1* (e.g., p.D90A in Scandinavian populations [26^▪^]), *TARDBP* (e.g., p.A382T in Sardinia [27]) have been linked to region-specific familial ALS clusters, often characterized by distinct clinical features. In the case of *SOD1*, despite considerable heterogeneity across different variants [28], *SOD1*-mediated ALS may be associated with a shorter disease duration compared to sporadic ALS. Similarly, pathogenic variants in *FUS* are typically linked to early-onset ALS with rapid progression and markedly reduced survival [29]. Beyond these, other ALS-associated genes – including *ALS2*, *DCTN1*, *MATR3*, *OPTN*, and *SETX* – have been associated with a more indolent disease trajectory [30].

ALS is now increasingly recognized as a multisystem disorder, with its variability extending beyond motor symptoms to include non-motor manifestations such as behavioral and cognitive changes, parkinsonism, and psychosis [30]. These observations support the view of ALS as a spectrum disease, whose phenotypic expression is shaped by a dynamic interplay of genetic architecture, environmental context, and population ancestry [30].

## WHAT WE KNOW ABOUT ALS IN EGYPT AND ARABIC COUNTRIES: THE EGYPTIAN REGISTRY PROJECT

Comprehensive epidemiological data on ALS in Egypt are limited by the absence of large-scale population-based studies. A proposal has been made to establish a population-based registry for ALS and MNDs in Egypt [12^▪▪^]. This initiative provides a platform to investigate environmental exposures and gene-environment interactions that may contribute to ALS development, in a country with a population exceeding 100 million, and enhances the potential for ALS patients in Egypt – and Africa more broadly – to access modern individualized genetic therapies. The registry adopts a reconstructed cohort design, focusing on five Egyptian governorates: Cairo, Alexandria, Dakahlia, Assiut, and Gharbia. These regions were selected for their urban infrastructure, access to specialized neuromuscular centers, and comprehensive demographic data from Egypt's Central Agency for Public Mobilization and Statistics (CAPMAS). Standardized clinical and epidemiological protocols are being utilized for data collection. Moreover, Arabic/Egyptian versions of the ALS Functional Rating Scale (ALSFRS-R) and the Edinburgh Cognitive and Behavioural ALS Screen (ECAS) have been validated for use within the registry [31].

Preliminary findings from the Neuromuscular Unit of Ain Shams University Hospital, which recruited 203 ALS patients [32^▪^], report that 18% of cases are familial, with a male-to-female ratio of 2.5 : 1. Additionally 19% of cases are juvenile ALS (onset before 25  years). The mean age at onset for classic ALS is 39  years, which is lower than estimates for other African and North African countries [33], possibly reflecting genetic and environmental factors unique to the region. The median diagnostic delay is 12  months, with 76% of cases having spinal onset and 24% bulbar onset. Regarding treatment, 64% of patients received riluzole, while 20% received both riluzole and edaravone [32^▪^].

Recent genetic studies on familial ALS in Egypt suggest early disease onset, high rates of consanguinity, and specific genetic variations that may influence clinical presentation and disease progression [34,35]. Among familial ALS patients in Egypt, *SOD1* variants are the most frequent [34], raising hope for the introduction and development of modern individualized genetic therapies for this population.

## GLOBAL BURDEN OF DISEASE STUDY RESULTS FOR ALS IN EGYPT AND ARABIC MEDITERRANEAN COUNTRIES

**FIGURE 1:**
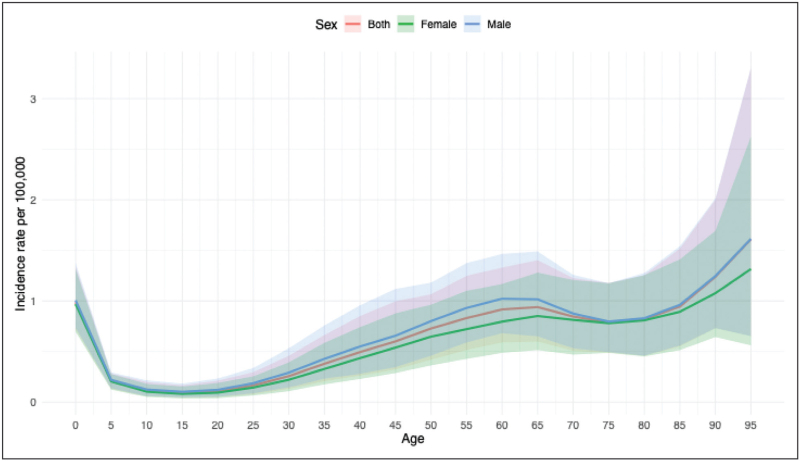
Incidence rate per 100 000 by age and sex for Egypt in 2021.

According to the GBD study, in 2021, Egypt reported 2268.28 cases (95% uncertainty interval (UI), 362.49–514.76) of MND, which equates to 2.16 age-standardized cases per 100 000 people (95% UI, 1.73–2.62; Table 1). MND in Egypt exhibited a triphasic incidence pattern: before five years old, around 60  years, and after 95 years respectively, without sex differences (Fig. 1). Between 1990 and 2021, the absolute number of new MND cases increased by 78.0%, though age-standardized rates decreased by 9%. DALYs (disability-adjusted life years), YLLs (years of life lost) and deaths remained stable, but their age-standardized rates decreased by 48%, 99%, and 98%, respectively. In contrast, the absolute values of YLDs (years lived with disability) increased by 97%, although the age-standardized rates remained stable. Males and females show similar trends, except for DALYs, where females experienced a significant decreased in age-standardized rates (−57% [95% UI, −68 to −45]).

**Table 1:** Counts and age-standardized per 100 000 and changes between 1990 and 2021 for motor neuron disorders in Mediterranean and sub-Saharan region, both sexes

		Counts (95% uncertainty intervals)			Age-standardized (95% uncertainty intervals)		
		1990	2021	Change %	1990	2021	Change %
Prevalence	Egypt	1153.38 (919.04 to 1413.95)	2268.28 (2268.28 to 2761.9)	97.0	2.13 (1.72 to 2.59)	2.16 (1.73 to 2.62)	1.0
	North African Mediterranean^*^	847.62 (389.93 to 1076.2)	1580.79 (580.07 to 2086.46)	86.5	2.31 (2.17 to 2.58)	2.3 (2.19 to 2.53)	−0.5
	Turkey and the Levant^*^	1435.81 (131.85 to 1869.27)	2534.74 (215.71 to 3310.96)	76.5	3.64 (2.83 to 4.66)	3.99 (2.82 to 5.19)	9.5
	European Mediterranean^*^	4407.26 (430.55 to 5674.15)	8207.17 (766.73 to 10269.87)	86.2	7.07 (4.56 to 8.37)	8.99 (5.98 to 10.16)	27.0
	Sub-Saharan Africa	5817.3 (4415.0 to 7354.3)	13166.3 (9942.5 to 16752.1)	1.26	1.3 (1.0 to 1.7)	1.3 (1.0 to 1.6)	−0.05
Incidence	Egypt	246.87 (204.62 to 293.63)	438.21 (362.49 to 514.76)	78.0	0.50 (0.41 to 0.60)	0.45 (0.38 to 0.53)	−9.0
	North African Mediterranean^*^	172.35 (67.6 to 227.04)	299.68 (107.98 to 402.94)	73.9	0.49 (0.46 to 0.52)	0.45 (0.42 to 0.47)	−8.7
	Turkey and the Levant^*^	301.22 (25.19 to 392.07)	577.45 (37.23 to 759.72)	91.7	0.8 (0.55 to 0.94)	0.87 (0.5 to 0.99)	8.7
	European Mediterranean^*^	958.26 (80.98 to 1256.67)	1866.39 (176.67 to 2308.02)	94.8	1.48 (0.79 to 1.78)	1.85 (1.15 to 2.08)	24.6
	Sub-Saharan Africa	1673.5 (1383.7 to 2022.4)	3470.0 (2858.4 to 4220.6)	1.07	0.5 (0.4 to 0.6)	0.4 (0.3 to 0.5)	−0.11
DALYs	Egypt	550.30 (414.51 to 739.65)	488.41 (322.04 to 698.78)	−11.0	0.89 (0.68 to 1.17)	0.47 (0.31 to 0.66)	−48.0
	North African Mediterranean^*^	338.21 (84.47 to 496.89)	348.81 (135.37 to 452.02)	3.1	0.72 (0.53 to 0.86)	0.52 (0.48 to 0.6)	−27.9
	Turkey and the Levant^*^	7452.08 (81.49 to 10009.92)	16092.07 (157.26 to 21543.01)	115.9	14.95 (2.08 to 18.44)	22.78 (3.33 to 27.93)	52.4
	European Mediterranean^*^	20695.2 (1397.07 to 27332.62)	36698.31 (4104.91 to 45529.35)	77.3	35.05 (14.36 to 42.06)	38.97 (24.82 to 44.27)	11.2
	Sub-Saharan Africa	1702.3 (1235.5 to 2314.7)	3578.6 (2566.3 to 4976.0)	1.10	0.4 (0.3 to 0.5)	0.3 (0.3 to 0.5)	−0.10
YLDs	Egypt	245.35 (162.72 to 350.05)	482.56 (317.63 to 694.17)	97.0	0.45 (0.30 to 0.64)	0.46 (0.30 to 0.65)	1.0
	North African ^*^Mediterranean	180.3 (82.93 to 227.23)	336.3 (123.4 to 443.88)	86.5	0.49 (0.46 to 0.55)	0.49 (0.47 to 0.54)	−0.5
	Turkey and the Levant^*^	305.46 (27.68 to 396.88)	538.99 (45.88 to 703.79)	76.5	0.77 (0.6 to 0.99)	0.85 (0.59 to 1.09)	9.5
	European Mediterranean^*^	936.47 (94.63 to 1205.32)	1738.23 (160.39 to 2174.37)	85.6	1.5 (0.96 to 1.78)	1.91 (1.29 to 2.15)	26.8
	Sub-Saharan Africa	1237.5 (800.9 to 1807.7)	2801.1 (1793.2 to 4089.5)	1.26	0.3 (0.2 to 0.4)	0.3 (0.2 to 0.4)	−0.05
YLLs	Egypt	304.96 (184.83 to 447.00)	5.84 (3.20 to 21.91)	−98.0	0.44 (0.26 to 0.62)	0.006 (0.003 to 0.024)	−99.0
	North African^*^ Mediterranean	157.91 (1.54 to 272.11)	12.51 (5.98 to 23.68)	−92.1	0.23 (0.01 to 0.39)	0.03 (0.01 to 0.07)	−85.9
	Turkey and the Levant^*^	7146.63 (39.7 to 9593.87)	15553.07 (99.48 to 20840.67)	117.6	14.17 (1.53 to 17.64)	21.94 (2.58 to 27.07)	54.8
	European Mediterranean^*^	19758.73 (1171.9 to 26091.06)	34960.08 (3970.07 to 43511.33)	76.9	33.55 (13.12 to 40.15)	37.06 (24.64 to 42.13)	10.5
	Sub-Saharan Africa	464.8 (269.2 to 705.8)	777.5 (482.9 to 1096.9)	0.67	0.1 (0.1 to 0.2)	0.1 (0.1 to 0.1)	−0.23
Deaths	Egypt	4.38 (2.61 to 6.18)	0.12 (0.07 to 0.50)	−97.0	0.008 (0.005 to 0.011)	0.0002 (0.0001 to 0.0006)	−98.0
	North African Mediterranean^*^	2.27 (0.03 to 3.91)	0.31 (0.13 to 0.59)	−86.4	0 (0 to 0.01)	0 (0 to 0)	−79.7
	Turkey and the Levant^*^	114.47 (0.9 to 151.86)	430.11 (3.17 to 572.4)	275.8	0.3 (0.04 to 0.49)	0.57 (0.08 to 0.7)	92.6
	European Mediterranean^*^	712.07 (46.71 to 980.09)	1571.35 (169.23 to 1943.75)	120.7	1.02 (0.39 to 1.29)	1.36 (0.91 to 1.54)	33.3
	Sub-Saharan Africa	8.7 (4.4 to 12.4)	14.8 (9.3 to 21.0)	0.71	0.0 (0.0 to 0.0)	0.0 (0.0 to 0.0)	−0.28

DALYS, disability-adjusted life years; YLDs, years lived with disability; YLLs, years of life lost.

* Regional estimates not provided by the Global Burden of Disease study 2021. Estimates correspond to weighted means using countries motor neuron disease Global burden of disease study 2021 estimates and weighting them by population size. Uncertainty intervals were calculated using bootstrapping (10 000 samples).

**FIGURE 2:**
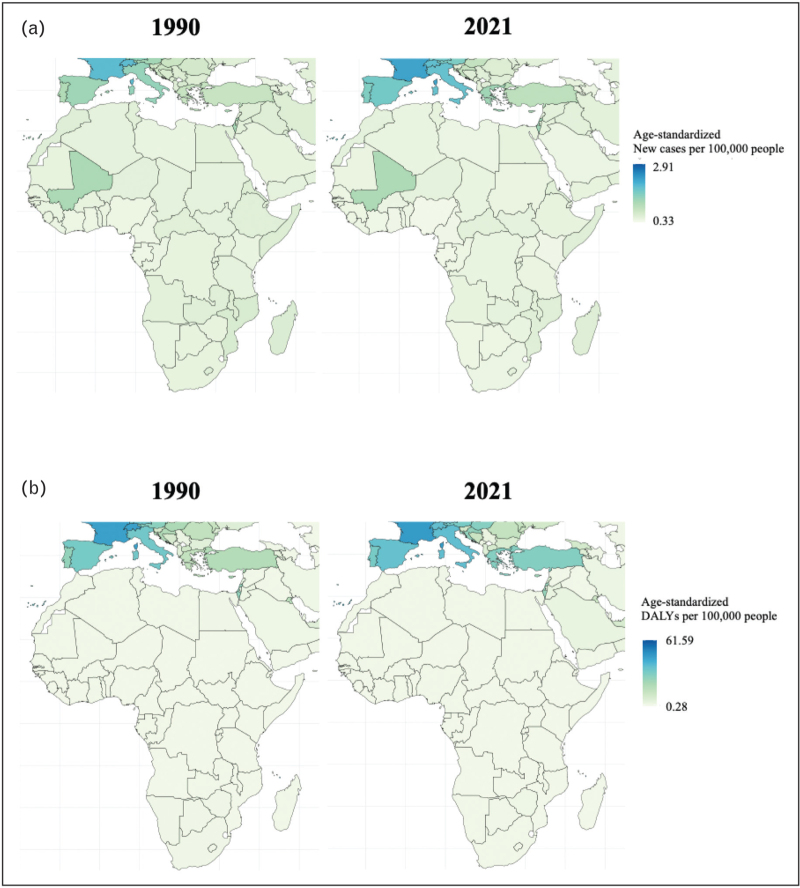
Age-standardized incidence and DALYs in the Mediterranean area and Africa, in 1990 and 2021 for both sexes.

**FIGURE 3:**
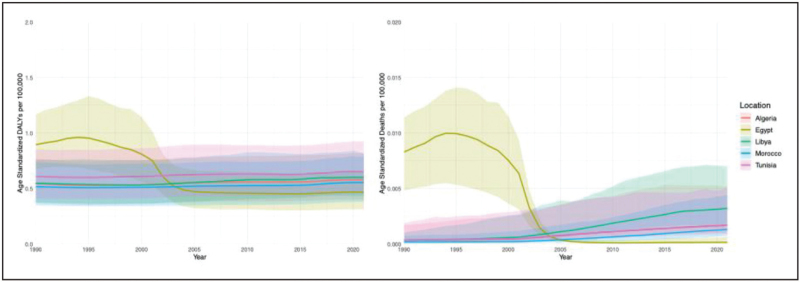
Age-standardized DALYs and deaths from 1990 to 2021 in African Mediterranean countries, both sexes.

In the African Mediterranean region, there were 1580.79 reported cases of MND in 2021. Comparing Egypt with African Mediterranean countries, Egypt exhibited age-standardized rates comparable to those of neighboring countries in the subregion, including Morocco, Algeria, Tunisia, and Libya (Fig. 2, Table 1, Supplemental Digital Content). Nevertheless, between 1990 and 2021, Egypt was the only country in the region with a decline in both MND DALYs and deaths, while these metrics increased in other countries (Fig. 3). Trends in incidence and prevalence were similar across African Mediterranean countries. No significant sex differences were observed, except for DALYs, where females in Egypt experienced a consistent decrease compared to their counterparts in neighboring countries.

In the Mediterranean Levant region and Turkey there were 2543.74 cases of MND in 2021. Among these countries, Egypt, had the lowest age-standardized prevalence rate, while Israel reported the highest at 5.85 per 100 000 people (95% UI, 4.99–6.77). Likewise, Israel had the largest age-standardized incidence rate at 1.21 (95% UI, 1.11–1.31), while Egypt, along with Palestine and Lebanon, recorded the lowest rates. In contrast, Turkey reported the highest age-standardized DALYs (17.97 [95% UI, 10.65–31.35]) and deaths (0.32 [95% UI, 0.18–0.55), while Egypt, the lowest values for both. Between 1990 and 2021, Egypt was the only country in this region to experience a decrease in age-standardized DALYs and deaths. For prevalence and incidence, Egypt showed trends similar to Lebanon and Palestine. Israel, Cyprus and Turkey followed a shared pattern of increasing rates. Across most countries in the region, males had higher age-standardized rates than females for incidence, prevalence, DALYs and deaths. A similar pattern was seen in Cyprus, and Turkey (for incidence only), whereas in Palestine, females had higher age-standardized DALYs and deaths. Between 1990 and 2021, males in Turkey showed a consistent increase in incidence rates, while rates among females remained stable. In Israel, DALYs increased among males but decreased among females; similarly, mortality rates rose only among males.

In the European Mediterranean region, there were 8207.17 MND cases reported in 2021. France recorded the highest prevalence in the region (10.84 [95% UI, 9.52–12.42]). Incidence rates were relatively homogeneous across the region. Egypt trends were more similar to those observed in Montenegro, Slovenia, Bosnia and Herzegovina, and Albania. In terms of age-standardized DALYs and deaths, Egypt had significantly lower rates than all European Mediterranean countries, with France reporting the highest. From 1990 to 2021, Egypt maintained stable age-standardized prevalence rates, similar to Bosnia and Herzegovina, Albania, and Montenegro. In contrast, Greece recorded the largest consistent increase in prevalence (45%), while Slovenia showed the greatest decrease (−13%). A similar trend was observed for incidence, with Greece showing a 56% increase and Slovenia a 28% decrease. For DALYs and deaths, Egypt was the only country to show a decline. In contrast, Greece exhibited the largest increases in DALYs (116%) and deaths (151%) in the region. Regarding sex differences, most countries had higher age-standardized rates in males than females, except for Montenegro and Monaco, where females had higher age-standardized DALYs and deaths.

## COMPARISON WITH OTHER AREAS OF AFRICA

In 2021, Sub-Saharan Africa recorded 13166.3 cases of MND. Compared to countries in this macro-region, Egypt had the highest age-standardized prevalence rate. However, its age-standardized incidence, DALYs and death rate were comparable to those observed in other Sub-Saharan countries. Between 1990 to 2021, Egypt maintained stable age-standardized prevalence rates, similar to most Sub-Saharan African countries. Only São Tomé and Príncipe and Equatorial Guinea experienced increases in prevalence over this period. For incidence, São Tomé and Príncipe was the only country to report a significant increase (33%) while Egypt, like the majority of Sub-Saharan countries, showed a decline. Notably, in terms of DALYs and deaths, Egypt experienced the largest decrease in age-standardized rates among African counties. In contrast, São Tomé and Príncipe showed the highest increases- 39% for DALYs and 151% for deaths.

## CONCLUSION

The epidemiology of ALS in Arabic Mediterranean countries, particularly in Egypt, presents a unique pattern, with lower age-standardized incidence, prevalence, and disease burden compared to both Southern Europe and the Levant region. This variability likely reflects differences in genetic background, demographic structure, and healthcare access.

The establishment of a national ALS registry in Egypt marks a significant step forward in strengthening epidemiological data collection in the region. According to the most recent GBD estimates, Egypt stands out for its decline in both DALYs and mortality related to ALS over the past three decades, contrasting with the increasing trends in neighboring countries. This study, utilizing GBD data, confirms the global heterogeneity of ALS frequencies across different geographic regions [2,36].

These findings underscore the importance of expanding collaborative research and registry-based studies across Arabic countries to improve our understanding of ALS and to enhance public health responses.

## Acknowledgements

*None.*

### Financial support and sponsorship

*This work has been supported with the founding of Regione Puglia and CNR for Tecnopolo per la Medicina di Precisione. D.G.R. no. 2117 of 21.11.2018 (B84I18000540002).*

### Conflicts of interest

*There are no conflicts of interest.*

## Supplementary Material

Supplemental Digital Content

## REFERENCES AND RECOMMENDED READING

Papers of particular interest, published within the annual period of review, have been highlighted as:

▪ of special interest
▪▪ of outstanding interest
