# Supplementary material for: Heterogeneity of frequencies of motor neuron disease across ethnicities and geographical areas: focus on Arabic countries in the Mediterranean area
Source: Curr Opin Neurol. 2025 Aug 22;38(5):588–95. doi: 10.1097/WCO.0000000000001415 (PMC12708033; doi:10.1097/WCO.0000000000001415)
Supplement: Supplemental Digital Content [file coneu-38-588-s001.docx]

**Supplemental Material**

**Supplemental Table 1.** Counts and Age-standardized per 100,000 and changes between 1990 and 2021 for motor neuron disorders in Mediterranean and Sub-Saharan regions, both sexes.

|  |  | **Counts** | | | **Age-standardized** | | |
| --- | --- | --- | --- | --- | --- | --- | --- |
|  |  | **1990** | **2021** | **Change** | **1990** | **2021** | **Change** |
| North African Mediterranean |  |  |  |  |  |  |  |
| Egypt | Prevalence | 1153.38  (919.04 to 1413.95) | 2268.28  (362.49 to 514.76) | 97.0  (87.0 to 107.0) | 2.13  (1.72 to 2.59) | 2.16  (1.73 to 2.62) | 1.0  (-3.0 to 6.0) |
|  | Incidence | 246.87  (204.62 to 293.63) | 438.21  (362.49 to 514.76) | 78.0 (66.0 to 87.0) | 0.50 (0.41 to 0.60) | 0.45 (0.38 to 0.53) | -9.0  (-14.0 to  -4.0) |
|  | DALYs | 550.30  (414.51 to 739.65) | 488.41  (322.04 to 698.78) | -11.0 (-35.0 to 18.0) | 0.89 (0.68 to 1.17) | 0.47 (0.31 to 0.66) | -48.0 (-60.0 to  -33.0) |
|  | YLDs | 245.35  (162.72 to 350.05) | 482.56 (317.63 to 694.17) | 97.0 (87.0 to 107.0) | 0.45 (0.30 to 0.64) | 0.46 (0.30 to 0.65) | 1.0 (-3.0 to 6.0) |
|  | YLLs | 304.96 (184.83 to 447.00) | 5.84 (3.20 to 21.91) | -98.0  (-99.0 to -90.0) | 0.44 (0.26 to 0.62) | 0.006 (0.003 to 0.024) | -99.0 (-99.0 to  -93.0) |
|  | Deaths | 4.38 (2.61 to 6.18) | 0.12 (0.07 to 0.50) | -97.0 (-98.0 to -85.0) | 0.008 (0.005 to 0.011) | 0.0002 (0.0001 to 0.0006) | -98.0 (-99.0 to  -89.0) |
| Libya | Prevalence | 102.31 (80.36 to 126.75) | 160.97 (127.7 to 197.11) | 57.0 (42.0 to 72.0) | 2.51 (2.02 to 3.06) | 2.27 (1.81 to 2.74) | -10.0 (-14.0 to -6.0) |
|  | Incidence | 15.15 (12.76 to 18.22) | 25.2 (20.49 to 30.45) | 66.0 (46.0 to 87.0) | 0.42 (0.35 to 0.51) | 0.4 (0.33 to 0.47) | -6.0 (-10.0 to -2.0) |
|  | DALYs | 22.26 (15.16 to 31.48) | 41.09 (29.09 to 56.93) | 85.0 (64.0 to 127.0) | 0.55 (0.37 to 0.76) | 0.6 (0.43 to 0.82) | 10.0 (-1.0 to 35.0) |
|  | YLDs | 21.76 (14.65 to 31.06) | 34.24 (22.59 to 48.32) | 57.0 (42.0 to 72.0) | 0.53 (0.36 to 0.75) | 0.48 (0.33 to 0.68) | -10.0 (-14.0 to -6.0) |
|  | YLLs | 0.5 (0.24 to 1.6) | 6.85 (3.89 to 15.95) | 1277.0 (525.0 to 2519.0) | 0.01 (0.01 to 0.04) | 0.12 (0.07 to 0.26) | 844.0 (300.0 to 1637.0) |
|  | Deaths | 0.01 (0 to 0.03) | 0.18 (0.1 to 0.41) | 1909.0 (789.0 to 3568.0) | 0 (0 to 0) | 0 (0 to 0.01) | 919.0 (344.0 to 1789.0) |
| Tunisia | Prevalence | 229.7 (183.08 to 278.36) | 332.72 (271.44 to 401.73) | 45.0 (34.0 to 57.0) | 2.79 (2.28 to 3.37) | 2.78 (2.24 to 3.35) | 0.0 (-5.0 to 4.0) |
|  | Incidence | 37.08 (31.1 to 43.57) | 55.45 (45.35 to 67.2) | 50.0 (36.0 to 63.0) | 0.51 (0.42 to 0.61) | 0.45 (0.38 to 0.54) | -10.0 (-15.0 to -7.0) |
|  | DALYs | 50.16 (33.67 to 71.44) | 78.03 (53.83 to 110.45) | 56.0 (42.0 to 80.0) | 0.61 (0.41 to 0.85) | 0.65 (0.45 to 0.92) | 7.0 (0.0 to 22.0) |
|  | YLDs | 48.86 (32.54 to 69.97) | 70.77 (47.23 to 98.54) | 45.0 (34.0 to 57.0) | 0.59 (0.39 to 0.84) | 0.59 (0.4 to 0.84) | 0.0 (-5.0 to 4.0) |
|  | YLLs | 1.3 (0.57 to 5.62) | 7.26 (3.59 to 23.12) | 459.0 (195.0 to 1019.0) | 0.02 (0.01 to 0.07) | 0.06 (0.03 to 0.19) | 281.0 (100.0 to 650.0) |
|  | Deaths | 0.02 (0.01 to 0.12) | 0.22 (0.12 to 0.66) | 840.0 (416.0 to 1873.0) | 0 (0 to 0) | 0 (0 to 0.01) | 386.0 (151.0 to 974.0) |
| Algeria | Prevalence | 608.02 (480.64 to 746.64) | 1071.81 (854.76 to 1307.95) | 76.0 (64.0 to 92.0) | 2.49 (2.03 to 3.02) | 2.43 (1.94 to 2.96) | -3.0 (-7.0 to 2.0) |
|  | Incidence | 100.26 (83.76 to 118.12) | 184.05 (150.19 to 221.96) | 84.0 (68.0 to 98.0) | 0.46 (0.38 to 0.55) | 0.42 (0.35 to 0.51) | -7.0 (-11.0 to -4.0) |
|  | DALYs | 133.31 (90.43 to 191.1) | 255.06 (175.22 to 359.25) | 91.0 (75.0 to 122.0) | 0.55 (0.37 to 0.77) | 0.58 (0.4 to 0.82) | 6.0 (0.0 to 22.0) |
|  | YLDs | 129.33 (87.33 to 184.74) | 228.01 (151.92 to 324.01) | 76.0 (64.0 to 92.0) | 0.53 (0.36 to 0.74) | 0.52 (0.34 to 0.73) | -3.0 (-7.0 to 2.0) |
|  | YLLs | 3.98 (1.73 to 18.36) | 27.05 (15.6 to 85.8) | 580.0 (262.0 to 1159.0) | 0.01 (0.01 to 0.07) | 0.06 (0.04 to 0.2) | 328.0 (136.0 to 665.0) |
|  | Deaths | 0.06 (0.03 to 0.32) | 0.66 (0.38 to 2.03) | 918.0 (470.0 to 1689.0) | 0 (0 to 0) | 0 (0 to 0.01) | 408.0 (197.0 to 828.0) |
| Morocco | Prevalence | 2.54 (2.12 to 3.07) | 3.83 (3.21 to 4.56) | 49.0 (39.0 to 60.0) | 2.39 (1.93 to 2.91) | 2.38 (1.93 to 2.89) | -1.0 (-5.0 to 5.0) |
|  | Incidence | 0.38 (0.33 to 0.44) | 0.55 (0.48 to 0.62) | 45.0 (34.0 to 58.0) | 0.53 (0.44 to 0.65) | 0.47 (0.39 to 0.57) | -10.0 (-14.0 to -6.0) |
|  | DALYs | 3.54 (2.63 to 4.66) | 8.51 (6.27 to 11.48) | 60.0 (47.0 to 88.0) | 0.52 (0.35 to 0.74) | 0.55 (0.38 to 0.78) | 7.0 (0.0 to 27.0) |
|  | YLDs | 0.54 (0.37 to 0.73) | 0.81 (0.56 to 1.08) | 49.0 (39.0 to 60.0) | 0.51 (0.34 to 0.72) | 0.51 (0.34 to 0.71) | -1.0 (-5.0 to 5.0) |
|  | YLLs | 3 (2.04 to 4.09) | 7.7 (5.44 to 10.6) | 709.0 (375.0 to 1253.0) | 0.01 (0 to 0.03) | 0.05 (0.02 to 0.17) | 510.0 (273.0 to 913.0) |
|  | Deaths | 0.13 (0.09 to 0.18) | 0.35 (0.25 to 0.48) | 1197.0 (738.0 to 2061.0) | 0 (0 to 0) | 0 (0 to 0) | 663.0 (379.0 to 1259.0) |
| Turkey and the Levant |  |  |  |  |  |  |  |
| Turkey | Prevalence | 2001.62 (1672.39 to 2364.07) | 3548.16 (3010.35 to 4136.76) | 77.0 (63.0 to 92.0) | 3.64 (3.06 to 4.28) | 4.06 (3.47 to 4.7) | 11.0 (6.0 to 18.0) |
|  | Incidence | 422.29 (384.35 to 462.03) | 814.93 (747.19 to 887.89) | 93.0 (84.0 to 103.0) | 0.84 (0.76 to 0.92) | 0.95 (0.88 to 1.03) | 13.0 (9.0 to 18.0) |
|  | DALYs | 10744.76 (6198.89 to 19508.13) | 23198.34 (16443.8 to 32617.33) | 116.0 (11.0 to 298.0) | 17.97 (10.65 to 31.35) | 29.07 (20.47 to 40.42) | 62.0 (-16.0 to 192.0) |
|  | YLDs | 425.84 (289.28 to 589.63) | 754.5 (525.79 to 1004.99) | 77.0 (63.0 to 92.0) | 0.77 (0.53 to 1.05) | 0.86 (0.6 to 1.15) | 11.0 (6.0 to 18.0) |
|  | YLLs | 10318.92 (5794.41 to 19053.38) | 22443.84 (15677.15 to 31927.45) | 118.0 (10.0 to 312.0) | 17.2 (9.95 to 30.52) | 28.2 (19.43 to 39.45) | 64.0 (-16.0 to 203.0) |
|  | Deaths | 162.92 (92.84 to 282.57) | 616.41 (411.59 to 891.82) | 278.0 (97.0 to 612.0) | 0.32 (0.18 to 0.55) | 0.7 (0.48 to 0.99) | 115.0 (16.0 to 309.0) |
| Syria | Prevalence | 325.51 (258.79 to 400.31) | 357.7 (288.87 to 438.62) | 10.0 (1.0 to 21.0) | 2.66 (2.18 to 3.25) | 2.56 (2.06 to 3.12) | -4.0 (-8.0 to 1.0) |
|  | Incidence | 59 (49.71 to 70.96) | 65.65 (53.71 to 80.26) | 11.0 (-4.0 to 27.0) | 0.54 (0.44 to 0.65) | 0.49 (0.4 to 0.59) | -9.0 (-13.0 to -5.0) |
|  | DALYs | 70.95 (47.65 to 100.69) | 82.32 (56.23 to 116.53) | 16.0 (6.0 to 31.0) | 0.58 (0.39 to 0.82) | 0.59 (0.41 to 0.83) | 2.0 (-4.0 to 13.0) |
|  | YLDs | 69.25 (45.93 to 99.33) | 76.08 (50.88 to 107.99) | 10.0 (1.0 to 21.0) | 0.57 (0.37 to 0.8) | 0.54 (0.37 to 0.77) | -4.0 (-8.0 to 1.0) |
|  | YLLs | 1.69 (0.7 to 8.24) | 6.24 (3.1 to 19.59) | 268.0 (73.0 to 654.0) | 0.01 (0.01 to 0.06) | 0.05 (0.02 to 0.14) | 281.0 (81.0 to 669.0) |
|  | Deaths | 0.03 (0.01 to 0.13) | 0.18 (0.09 to 0.55) | 550.0 (218.0 to 1237.0) | 0 (0 to 0) | 0 (0 to 0) | 353.0 (159.0 to 875.0) |
| Lebanon | Prevalence | 88.81 (71.67 to 107.2) | 168.24 (135.72 to 202.57) | 89.0 (77.0 to 101.0) | 3.01 (2.46 to 3.6) | 2.95 (2.39 to 3.54) | -2.0 (-6.0 to 3.0) |
|  | Incidence | 13.68 (11.36 to 16.15) | 24.22 (19.86 to 29.27) | 77.0 (64.0 to 89.0) | 0.48 (0.4 to 0.58) | 0.44 (0.37 to 0.53) | -8.0 (-12.0 to -4.0) |
|  | DALYs | 72.29 (41.95 to 126.46) | 264.25 (175.18 to 341.53) | 266.0 (124.0 to 519.0) | 2.38 (1.38 to 4.13) | 4.97 (3.29 to 6.61) | 108.0 (27.0 to 248.0) |
|  | YLDs | 18.89 (12.78 to 26.51) | 35.79 (23.52 to 50.17) | 89.0 (77.0 to 101.0) | 0.64 (0.43 to 0.9) | 0.63 (0.42 to 0.88) | -2.0 (-6.0 to 3.0) |
|  | YLLs | 53.4 (24.68 to 107.93) | 228.46 (138.38 to 305.06) | 328.0 (130.0 to 768.0) | 1.74 (0.78 to 3.51) | 4.34 (2.67 to 5.87) | 149.0 (31.0 to 408.0) |
|  | Deaths | 1.12 (0.49 to 2.25) | 6.5 (3.93 to 8.63) | 482.0 (202.0 to 1141.0) | 0.04 (0.02 to 0.09) | 0.12 (0.07 to 0.15) | 173.0 (37.0 to 497.0) |
| Israel | Prevalence | 286.08  (244.6 to 331.33) | 713.41 (612.56 to 814.3) | 149.0 (135.0 to 167.0) | 5.85 (4.99 to 6.77) | 6.65 (5.76 to 7.64) | 14.0 (7.0 to 20.0) |
|  | Incidence | 58.38  (53.68 to 63.43) | 143.25 (133.43 to 152.8) | 145.0 (136.0 to 155.0) | 1.21 (1.11 to 1.31) | 1.28 (1.19 to 1.37) | 6.0 (2.0 to 10.0) |
|  | DALYs | 1267.28  (1209.33 to 1325.58) | 2835.95 (2562.9 to 3114.25) | 124.0 (101.0 to 148.0) | 25.93 (24.78 to 27.07) | 25.29 (22.86 to 27.72) | -2.0 (-12.0 to 8.0) |
|  | YLDs | 60.79  (42.69 to 82.46) | 151.57 (107.46 to 204.29) | 149.0 (135.0 to 167.0) | 1.24 (0.87 to 1.69) | 1.41 (0.99 to 1.91) | 14.0 (7.0 to 20.0) |
|  | YLLs | 1206.48  (1150.06 to 1263.87) | 2684.38 (2406.96 to 2961.58) | 122.0 (99.0 to 148.0) | 24.69 (23.6 to 25.82) | 23.88 (21.54 to 26.21) | -3.0 (-13.0 to 8.0) |
|  | Deaths | 38.31  (36.21 to 40.21) | 109.5 (96.44 to 121.41) | 186.0 (154.0 to 220.0) | 0.78 (0.74 to 0.81) | 0.91 (0.8 to 1) | 17.0 (4.0 to 30.0) |
| Palestine | Prevalence | 50.54  (39.87 to 61.82) | 133.7 (106.93 to 164.95) | 165.0 (147.0 to 180.0) | 2.6 (2.1 to 3.14) | 2.58 (2.06 to 3.12) | -1.0 (-5.0 to 4.0) |
|  | Incidence | 9.83  (8.16 to 11.64) | 21.41 (17.71 to 25.29) | 118.0 (100.0 to 135.0) | 0.54 (0.44 to 0.66) | 0.49 (0.41 to 0.59) | -9.0 (-12.0 to -5.0) |
|  | DALYs | 38.56  (26.94 to 54.87) | 134.65 (102.57 to 179.64) | 249.0 (124.0 to 421.0) | 1.59 (1.18 to 2.29) | 2.96 (2.31 to 3.74) | 86.0 (33.0 to 160.0) |
|  | YLDs | 10.75  (7.21 to 15.42) | 28.44 (19.05 to 40.62) | 165.0 (147.0 to 180.0) | 0.55 (0.37 to 0.79) | 0.55 (0.36 to 0.78) | -1.0 (-5.0 to 4.0) |
|  | YLLs | 27.81  (17.42 to 44.08) | 106.21 (76.99 to 148.36) | 282.0 (112.0 to 572.0) | 1.03 (0.68 to 1.67) | 2.41 (1.81 to 3.18) | 133.0 (46.0 to 270.0) |
|  | Deaths | 0.41  (0.27 to 0.65) | 2.08 (1.58 to 2.71) | 412.0 (209.0 to 698.0) | 0.02 (0.01 to 0.04) | 0.06 (0.05 to 0.08) | 176.0 (67.0 to 349.0) |
| Cyprus | Prevalence | 45.95  (39.4 to 53.68) | 112.05 (95.67 to 131.4) | 144.0 (130.0 to 163.0) | 5.58 (4.76 to 6.5) | 6.38 (5.46 to 7.37) | 14.0 (8.0 to 21.0) |
|  | Incidence | 9.33  (8.44 to 10.2) | 22.53 (20.9 to 24.26) | 141.0 (130.0 to 153.0) | 1.14 (1.03 to 1.24) | 1.21 (1.11 to 1.3) | 6.0 (2.0 to 11.0) |
|  | DALYs | 155.3  (71.36 to 254.21) | 446.02 (293.13 to 610.9) | 187.0 (72.0 to 499.0) | 19.77 (9.12 to 32.05) | 23.42 (15.5 to 32.03) | 18.0 (-29.0 to 149.0) |
|  | YLDs | 9.76  (6.8 to 13.27) | 23.81 (16.85 to 31.88) | 144.0 (130.0 to 163.0) | 1.19 (0.83 to 1.6) | 1.36 (0.96 to 1.82) | 14.0 (8.0 to 21.0) |
|  | YLLs | 145.54  (61.72 to 244.47) | 422.21 (265.99 to 586.16) | 190.0 (70.0 to 554.0) | 18.59 (7.94 to 30.65) | 22.07 (14.18 to 30.61) | 19.0 (-31.0 to 169.0) |
|  | Deaths | 5.59  (2.32 to 9.63) | 18.03 (11.41 to 24.85) | 222.0 (82.0 to 650.0) | 0.74 (0.3 to 1.28) | 0.87 (0.55 to 1.19) | 18.0 (-33.0 to 181.0) |
| European Mediterranean |  |  |  |  |  |  |  |
| Spain | Prevalence | 2991.3 (2575.13 to 3451) | 5937.15 (5089.65 to 6962.4) | 98.0 (82.0 to 119.0) | 6.32 (5.45 to 7.3) | 8.23 (7.13 to 9.51) | 30.0 (21.0 to 41.0) |
|  | Incidence | 621.49 (579.31 to 668.75) | 1333.08 (1262.95 to 1408.38) | 114.0 (106.0 to 124.0) | 1.29 (1.2 to 1.39) | 1.69 (1.59 to 1.78) | 31.0 (26.0 to 37.0) |
|  | DALYs | 13919.64 (13274.6 to 14585.55) | 27442.24 (23641.85 to 32065.22) | 97.0 (68.0 to 132.0) | 31.85 (30.44 to 33.36) | 36.01 (31.43 to 41.58) | 13.0 (-3.0 to 32.0) |
|  | YLDs | 635.5 (455.99 to 865.46) | 1260.96 (911.13 to 1673.42) | 98.0 (82.0 to 119.0) | 1.34 (0.96 to 1.84) | 1.75 (1.27 to 2.32) | 30.0 (21.0 to 41.0) |
|  | YLLs | 13284.14 (12615.6 to 13913.53) | 26181.28 (22397.78 to 30755.88) | 97.0 (67.0 to 134.0) | 30.51 (29.11 to 31.91) | 34.26 (29.77 to 39.9) | 12.0 (-4.0 to 32.0) |
|  | Deaths | 461.7 (435.24 to 485.96) | 1153.56 (979.36 to 1359.8) | 150.0 (111.0 to 197.0) | 0.91 (0.86 to 0.95) | 1.28 (1.08 to 1.5) | 40.0 (19.0 to 66.0) |
| France | Prevalence | 6331.51 (5452.96 to 7243.22) | 11407.63 (9997.14 to 13107.04) | 80.0 (66.0 to 94.0) | 8.95 (7.76 to 10.17) | 10.84 (9.52 to 12.42) | 21.0 (13.0 to 28.0) |
|  | Incidence | 1406.71 (1327.68 to 1490.61) | 2543.86 (2428.29 to 2664.43) | 81.0 (74.0 to 87.0) | 1.93 (1.82 to 2.05) | 2.23 (2.11 to 2.35) | 15.0 (12.0 to 19.0) |
|  | DALYs | 30745.27 (29363.13 to 32019.75) | 50543.19 (43537.46 to 58162.59) | 64.0 (43.0 to 89.0) | 45.83 (44.19 to 47.64) | 47.4 (41.46 to 53.99) | 3.0 (-10.0 to 18.0) |
|  | YLDs | 1345.3 (962.1 to 1808.07) | 2409.62 (1719.35 to 3225.76) | 79.0 (65.0 to 93.0) | 1.9 (1.35 to 2.56) | 2.29 (1.64 to 3.08) | 21.0 (13.0 to 28.0) |
|  | YLLs | 29399.98 (28156.28 to 30617.27) | 48133.57 (41257.61 to 56039.32) | 64.0 (41.0 to 90.0) | 43.93 (42.28 to 45.68) | 45.1 (39.19 to 51.88) | 3.0 (-11.0 to 18.0) |
|  | Deaths | 1107 (1042.83 to 1160.3) | 2138.37 (1814.99 to 2510.88) | 93.0 (65.0 to 124.0) | 1.43 (1.36 to 1.49) | 1.65 (1.41 to 1.93) | 16.0 (0.0 to 34.0) |
| Monaco | Prevalence | 2.54 (2.12 to 3.07) | 3.83 (3.21 to 4.56) | 51.0 (43.0 to 62.0) | 5.93 (4.98 to 7.03) | 6.54 (5.55 to 7.62) | 10.0 (6.0 to 15.0) |
|  | Incidence | 0.38 (0.33 to 0.44) | 0.55 (0.48 to 0.62) | 44.0 (35.0 to 55.0) | 0.82 (0.7 to 0.93) | 0.83 (0.73 to 0.92) | 1.0 (-3.0 to 6.0) |
|  | DALYs | 3.54 (2.63 to 4.66) | 8.51 (6.27 to 11.48) | 140.0 (70.0 to 237.0) | 7.81 (5.9 to 10.25) | 12.98 (9.54 to 17.9) | 66.0 (18.0 to 128.0) |
|  | YLDs | 0.54 (0.37 to 0.73) | 0.81 (0.56 to 1.08) | 51.0 (43.0 to 62.0) | 1.26 (0.87 to 1.7) | 1.39 (0.96 to 1.88) | 10.0 (6.0 to 15.0) |
|  | YLLs | 3 (2.04 to 4.09) | 7.7 (5.44 to 10.6) | 156.0 (72.0 to 284.0) | 6.55 (4.62 to 8.9) | 11.59 (8.26 to 16.49) | 77.0 (19.0 to 161.0) |
|  | Deaths | 0.13 (0.09 to 0.18) | 0.35 (0.25 to 0.48) | 167.0 (79.0 to 303.0) | 0.21 (0.14 to 0.28) | 0.39 (0.28 to 0.54) | 89.0 (29.0 to 182.0) |
| Italy | Prevalence | 4720.7 (4050.13 to 5474.02) | 8959.87 (7624.06 to 10458.5) | 90.0 (78.0 to 103.0) | 6.49 (5.58 to 7.53) | 8.7 (7.48 to 10.02) | 34.0 (28.0 to 40.0) |
|  | Incidence | 1019.81 (946.51 to 1097.54) | 2109.52 (1992.32 to 2222.67) | 107.0 (100.0 to 115.0) | 1.38 (1.28 to 1.48) | 1.84 (1.74 to 1.96) | 33.0 (29.0 to 37.0) |
|  | DALYs | 21337.57 (20620.62 to 21960.85) | 39783.53 (35726.21 to 44492.75) | 86.0 (70.0 to 108.0) | 33.15 (32.19 to 34.06) | 37.51 (34.03 to 41.73) | 13.0 (3.0 to 26.0) |
|  | YLDs | 1003.19 (710.23 to 1339.93) | 1902.02 (1332.54 to 2574.73) | 90.0 (78.0 to 103.0) | 1.38 (0.96 to 1.86) | 1.85 (1.3 to 2.5) | 34.0 (29.0 to 40.0) |
|  | YLLs | 20334.38 (19691.1 to 20849.4) | 37881.51 (33971.43 to 42501.58) | 86.0 (68.0 to 109.0) | 31.77 (30.85 to 32.56) | 35.66 (32.24 to 39.98) | 12.0 (2.0 to 25.0) |
|  | Deaths | 693.1 (662.7 to 714.29) | 1749.35 (1529.54 to 1970.06) | 152.0 (127.0 to 181.0) | 0.88 (0.84 to 0.9) | 1.31 (1.17 to 1.47) | 49.0 (35.0 to 67.0) |
| Slovenia | Prevalence | 91.73 (77.04 to 107.81) | 83.73 (68.73 to 101.02) | -9.0 (-18.0 to 1.0) | 4.58 (3.86 to 5.39) | 3.97 (3.24 to 4.74) | -13.0 (-21.0 to -5.0) |
|  | Incidence | 14.14 (12.24 to 15.98) | 13.73 (11.12 to 16.9) | -3.0 (-14.0 to 10.0) | 0.7 (0.62 to 0.79) | 0.51 (0.42 to 0.6) | -28.0 (-35.0 to -21.0) |
|  | DALYs | 155.56 (145.35 to 166.42) | 261.88 (222.63 to 304.7) | 68.0 (43.0 to 98.0) | 7.66 (7.14 to 8.2) | 8.44 (7.25 to 9.69) | 10.0 (-6.0 to 28.0) |
|  | YLDs | 19.51 (13.42 to 26.52) | 17.8 (12.07 to 24.38) | -9.0 (-18.0 to 1.0) | 0.97 (0.67 to 1.33) | 0.84 (0.57 to 1.2) | -13.0 (-21.0 to -5.0) |
|  | YLLs | 136.05 (128.21 to 144.4) | 244.08 (204.8 to 287.89) | 79.0 (50.0 to 114.0) | 6.68 (6.28 to 7.12) | 7.6 (6.39 to 8.86) | 14.0 (-5.0 to 34.0) |
|  | Deaths | 3.97 (3.72 to 4.23) | 9.31 (7.86 to 10.99) | 135.0 (97.0 to 179.0) | 0.17 (0.16 to 0.19) | 0.24 (0.2 to 0.28) | 38.0 (17.0 to 64.0) |
| Croatia | Prevalence | 211.81 (179.07 to 246.97) | 216.75 (183.89 to 250.44) | 2.0 (-5.0 to 11.0) | 4.31 (3.66 to 5.02) | 4.64 (3.93 to 5.46) | 8.0 (2.0 to 15.0) |
|  | Incidence | 37.72 (32.89 to 42.42) | 51.51 (46.96 to 55.79) | 37.0 (27.0 to 48.0) | 0.74 (0.65 to 0.82) | 0.84 (0.77 to 0.92) | 14.0 (9.0 to 20.0) |
|  | DALYs | 918.94 (858.98 to 989.34) | 1619.89 (1362.33 to 1970.63) | 76.0 (46.0 to 120.0) | 17.75 (16.67 to 18.94) | 26.04 (22.1 to 31.28) | 47.0 (23.0 to 79.0) |
|  | YLDs | 45.04 (31.32 to 61.31) | 46.07 (32.19 to 61.16) | 2.0 (-5.0 to 11.0) | 0.92 (0.64 to 1.25) | 0.99 (0.69 to 1.34) | 8.0 (2.0 to 15.0) |
|  | YLLs | 873.91 (817.5 to 941.05) | 1573.82 (1314.57 to 1926.97) | 80.0 (49.0 to 127.0) | 16.84 (15.82 to 17.99) | 25.05 (20.94 to 30.27) | 49.0 (24.0 to 83.0) |
|  | Deaths | 25.55 (23.62 to 27.75) | 59.17 (49.54 to 73.44) | 132.0 (89.0 to 194.0) | 0.44 (0.41 to 0.48) | 0.76 (0.64 to 0.94) | 72.0 (42.0 to 115.0) |
| Bosnia and Herzegovina | Prevalence | 153.27 (125.18 to 181.91) | 117.26 (95.99 to 141.82) | -23.0 (-30.0 to -16.0) | 3.35 (2.76 to 3.96) | 3.55 (2.86 to 4.27) | 6.0 (-1.0 to 14.0) |
|  | Incidence | 26.31 (22.36 to 30.58) | 21.54 (17.15 to 26.51) | -18.0 (-27.0 to -8.0) | 0.62 (0.53 to 0.71) | 0.52 (0.43 to 0.62) | -16.0 (-22.0 to -11.0) |
|  | DALYs | 324.15 (179.74 to 429.92) | 505.38 (306.06 to 688.74) | 56.0 (7.0 to 126.0) | 7.33 (4.1 to 9.91) | 11.2 (6.87 to 16.2) | 53.0 (8.0 to 119.0) |
|  | YLDs | 32.6 (22.1 to 45.52) | 24.93 (16.68 to 34.18) | -24.0 (-30.0 to -16.0) | 0.71 (0.48 to 1) | 0.76 (0.51 to 1.07) | 6.0 (-1.0 to 14.0) |
|  | YLLs | 291.55 (148.11 to 398.06) | 480.45 (281.95 to 663.47) | 65.0 (10.0 to 149.0) | 6.62 (3.41 to 9.19) | 10.45 (6.14 to 15.43) | 58.0 (8.0 to 135.0) |
|  | Deaths | 7.32 (3.77 to 9.66) | 16.94 (9.97 to 23.07) | 131.0 (54.0 to 246.0) | 0.17 (0.09 to 0.22) | 0.3 (0.18 to 0.42) | 82.0 (22.0 to 169.0) |
| Montenegro | Prevalence | 21.8 (17.69 to 26.15) | 20.73 (17.09 to 25.13) | -5.0 (-11.0 to 1.0) | 3.45 (2.82 to 4.14) | 3.38 (2.77 to 4.05) | -2.0 (-6.0 to 3.0) |
|  | Incidence | 3.26 (2.65 to 3.95) | 3.53 (2.83 to 4.33) | 8.0 (1.0 to 14.0) | 0.53 (0.44 to 0.64) | 0.49 (0.41 to 0.59) | -7.0 (-11.0 to -4.0) |
|  | DALYs | 7.25 (5.69 to 9.32) | 8.38 (6.8 to 10.35) | 16.0 (-2.0 to 38.0) | 1.19 (0.95 to 1.52) | 1.2 (0.96 to 1.51) | 1.0 (-12.0 to 18.0) |
|  | YLDs | 4.64 (3.14 to 6.48) | 4.41 (2.98 to 6.09) | -5.0 (-11.0 to 1.0) | 0.73 (0.5 to 1.03) | 0.72 (0.48 to 1.02) | -2.0 (-6.0 to 3.0) |
|  | YLLs | 2.61 (1.83 to 3.37) | 3.98 (3.18 to 4.97) | 52.0 (5.0 to 134.0) | 0.46 (0.32 to 0.6) | 0.48 (0.38 to 0.61) | 5.0 (-27.0 to 56.0) |
|  | Deaths | 0.06 (0.04 to 0.08) | 0.14 (0.11 to 0.18) | 142.0 (66.0 to 282.0) | 0.01 (0.01 to 0.01) | 0.01 (0.01 to 0.02) | 54.0 (7.0 to 137.0) |
| Albania | Prevalence | 93.15 (75.31 to 113.01) | 81.72 (67.63 to 97.82) | -12.0 (-20.0 to -4.0) | 2.85 (2.32 to 3.44) | 2.94 (2.4 to 3.54) | 3.0 (-1.0 to 7.0) |
|  | Incidence | 15.56 (13.2 to 18.1) | 14.56 (12.28 to 17.05) | -6.0 (-16.0 to 4.0) | 0.52 (0.44 to 0.62) | 0.48 (0.41 to 0.56) | -8.0 (-12.0 to -4.0) |
|  | DALYs | 167.44 (112.01 to 237.56) | 118.59 (76.9 to 185.29) | -29.0 (-60.0 to 13.0) | 4.73 (3.26 to 6.61) | 4.73 (3.01 to 8.13) | 0.0 (-43.0 to 69.0) |
|  | YLDs | 19.82 (13.09 to 28.27) | 17.38 (11.59 to 23.73) | -12.0 (-20.0 to -4.0) | 0.61 (0.4 to 0.85) | 0.63 (0.41 to 0.87) | 3.0 (-1.0 to 7.0) |
|  | YLLs | 147.62 (93.13 to 216.46) | 101.21 (59.7 to 165.97) | -31.0 (-65.0 to 17.0) | 4.12 (2.66 to 6.01) | 4.1 (2.39 to 7.5) | 0.0 (-49.0 to 79.0) |
|  | Deaths | 2.14 (1.39 to 3.08) | 2.9 (1.71 to 4.63) | 36.0 (-27.0 to 126.0) | 0.07 (0.04 to 0.1) | 0.09 (0.05 to 0.14) | 25.0 (-31.0 to 103.0) |
| Greece | Prevalence | 565.68 (479.06 to 667.7) | 1032.19 (892.48 to 1195.29) | 82.0 (63.0 to 102.0) | 4.64 (3.93 to 5.44) | 6.74 (5.82 to 7.74) | 45.0 (33.0 to 59.0) |
|  | Incidence | 101.04 (88.31 to 114.32) | 218.04 (203.92 to 231.67) | 116.0 (96.0 to 139.0) | 0.82 (0.71 to 0.91) | 1.27 (1.18 to 1.36) | 56.0 (42.0 to 69.0) |
|  | DALYs | 1643.88 (1559.88 to 1725.9) | 5123.14 (4587.38 to 5728.65) | 212.0 (176.0 to 251.0) | 13.35 (12.73 to 13.92) | 28.84 (25.91 to 32.15) | 116.0 (92.0 to 142.0) |
|  | YLDs | 120.24 (84.06 to 161.59) | 219.28 (156.02 to 293.97) | 82.0 (63.0 to 102.0) | 0.99 (0.67 to 1.33) | 1.43 (1.02 to 1.94) | 45.0 (33.0 to 59.0) |
|  | YLLs | 1523.64 (1448.76 to 1591.25) | 4903.87 (4366.35 to 5514.36) | 222.0 (184.0 to 267.0) | 12.36 (11.82 to 12.87) | 27.41 (24.47 to 30.71) | 122.0 (95.0 to 151.0) |
|  | Deaths | 58.4 (55.15 to 61.65) | 219.08 (194.88 to 246.69) | 275.0 (232.0 to 325.0) | 0.4 (0.38 to 0.43) | 1.02 (0.9 to 1.14) | 151.0 (121.0 to 184.0) |
| Malta | Prevalence | 25.39 (21.87 to 29.47) | 55.08 (46.63 to 64.79) | 117.0 (98.0 to 139.0) | 6.24 (5.38 to 7.21) | 7.8 (6.74 to 8.99) | 25.0 (18.0 to 33.0) |
|  | Incidence | 5.65 (5.23 to 6.11) | 12.42 (11.66 to 13.22) | 120.0 (109.0 to 130.0) | 1.37 (1.27 to 1.47) | 1.55 (1.45 to 1.65) | 13.0 (9.0 to 17.0) |
|  | DALYs | 115.93 (108.32 to 123.43) | 266.32 (230.79 to 308.98) | 130.0 (97.0 to 169.0) | 28.83 (26.89 to 30.7) | 36.35 (31.36 to 42.14) | 26.0 (8.0 to 49.0) |
|  | YLDs | 5.39 (3.87 to 7.24) | 11.7 (8.38 to 15.95) | 117.0 (98.0 to 139.0) | 1.33 (0.95 to 1.77) | 1.66 (1.18 to 2.24) | 25.0 (18.0 to 33.0) |
|  | YLLs | 110.54 (103.14 to 117.46) | 254.62 (218.36 to 297.21) | 130.0 (96.0 to 171.0) | 27.5 (25.57 to 29.25) | 34.69 (29.58 to 40.44) | 26.0 (7.0 to 51.0) |
|  | Deaths | 4.07 (3.77 to 4.33) | 10.96 (9.33 to 12.8) | 170.0 (129.0 to 219.0) | 0.96 (0.89 to 1.02) | 1.21 (1.04 to 1.41) | 26.0 (7.0 to 49.0) |
| Sub-Saharan Africa |  |  |  |  |  |  |  |
| Western Sub-Sahara |  |  |  |  |  |  |  |
| Benin | Prevalence | 51.76 (39.56 to 66.11) | 147.45 (111.67 to 188.05) | 1.85 (1.73 to 1.99) | 1.23 (0.95 to 1.53) | 1.21 (0.95 to 1.5) | -0.02 (-0.05 to 0.02) |
|  | Incidence | 15.02 (12.45 to 18.15) | 37.95 (30.97 to 45.82) | 1.53 (1.4 to 1.64) | 0.41 (0.33 to 0.51) | 0.38 (0.3 to 0.46) | -0.08 (-0.11 to -0.05) |
|  | DALYs | 13.63 (9.63 to 18.98) | 38.84 (26.97 to 53.96) | 1.85 (1.59 to 2.17) | 0.31 (0.22 to 0.42) | 0.31 (0.23 to 0.43) | 0.02 (-0.06 to 0.11) |
|  | YLDs | 11.01 (7.27 to 16.22) | 31.37 (20.48 to 46.22) | 1.85 (1.73 to 1.99) | 0.26 (0.17 to 0.38) | 0.26 (0.17 to 0.37) | -0.02 (-0.05 to 0.02) |
|  | YLLs | 2.62 (1.55 to 4.27) | 7.47 (3.85 to 11.24) | 1.85 (0.74 to 3.25) | 0.05 (0.03 to 0.07) | 0.06 (0.03 to 0.08) | 0.2 (-0.26 to 0.75) |
|  | Deaths | 0.04 (0.02 to 0.06) | 0.12 (0.06 to 0.18) | 2.15 (1.01 to 3.61) | 0 (0 to 0) | 0 (0 to 0) | 0.23 (-0.23 to 0.81) |
| Burkina Faso | Prevalence | 99.87 (77.75 to 125.87) | 244.38 (188.31 to 314) | 1.45 (1.33 to 1.56) | 1.21 (0.94 to 1.5) | 1.19 (0.94 to 1.49) | -0.01 (-0.04 to 0.03) |
|  | Incidence | 32.68 (26.96 to 38.97) | 70.66 (58.05 to 85.23) | 1.16 (1.08 to 1.26) | 0.44 (0.36 to 0.55) | 0.41 (0.33 to 0.5) | -0.08 (-0.11 to -0.04) |
|  | DALYs | 24.24 (16.77 to 33.88) | 59.02 (40.89 to 83.03) | 1.43 (1.27 to 1.61) | 0.28 (0.2 to 0.39) | 0.29 (0.2 to 0.4) | 0 (-0.05 to 0.07) |
|  | YLDs | 21.24 (14.03 to 31.19) | 51.99 (33.92 to 76.34) | 1.45 (1.33 to 1.56) | 0.26 (0.17 to 0.36) | 0.25 (0.17 to 0.37) | -0.01 (-0.04 to 0.03) |
|  | YLLs | 3 (1.55 to 5.54) | 7.03 (3.3 to 12.6) | 1.34 (0.6 to 2.58) | 0.03 (0.01 to 0.05) | 0.03 (0.02 to 0.05) | 0.12 (-0.25 to 0.67) |
|  | Deaths | 0.05 (0.02 to 0.08) | 0.11 (0.06 to 0.2) | 1.52 (0.7 to 2.78) | 0 (0 to 0) | 0 (0 to 0) | 0.14 (-0.25 to 0.76) |
| Cabo Verde | Prevalence | 5.3 (4.15 to 6.6) | 9.39 (7.35 to 11.65) | 0.77 (0.63 to 0.92) | 1.63 (1.29 to 2.02) | 1.64 (1.29 to 2.02) | -0.03 (-0.07 to 0.01) |
|  | Incidence | 1.18 (0.97 to 1.4) | 1.87 (1.49 to 2.31) | 0.59 (0.44 to 0.75) | 0.42 (0.33 to 0.52) | 0.37 (0.29 to 0.45) | -0.07 (-0.1 to -0.04) |
|  | DALYs | 1.3 (0.92 to 1.8) | 2.51 (1.8 to 3.45) | 0.93 (0.71 to 1.25) | 0.4 (0.28 to 0.55) | 0.44 (0.32 to 0.6) | 0.02 (-0.09 to 0.16) |
|  | YLDs | 1.13 (0.75 to 1.64) | 2 (1.32 to 2.84) | 0.77 (0.63 to 0.92) | 0.35 (0.23 to 0.49) | 0.35 (0.24 to 0.49) | -0.03 (-0.07 to 0.01) |
|  | YLLs | 0.17 (0.09 to 0.33) | 0.51 (0.3 to 0.83) | 1.96 (0.63 to 4.26) | 0.05 (0.03 to 0.09) | 0.09 (0.05 to 0.15) | 0.24 (-0.28 to 0.88) |
|  | Deaths | 0 (0 to 0.01) | 0.01 (0.01 to 0.02) | 2.77 (1.06 to 5.65) | 0 (0 to 0) | 0 (0 to 0) | 0.25 (-0.27 to 0.95) |
| Cameroon | Prevalence | 108.28 (82.86 to 137.23) | 331.73 (252.53 to 425.82) | 2.06 (1.9 to 2.24) | 1.17 (0.91 to 1.45) | 1.12 (0.88 to 1.39) | 0.01 (-0.03 to 0.06) |
|  | Incidence | 30.46 (25.12 to 36.15) | 83.34 (68.87 to 100.25) | 1.74 (1.59 to 1.87) | 0.38 (0.31 to 0.46) | 0.35 (0.29 to 0.42) | -0.13 (-0.15 to -0.1) |
|  | DALYs | 32.97 (24.51 to 44.06) | 108.95 (79.88 to 148.32) | 2.3 (1.82 to 3.04) | 0.34 (0.26 to 0.45) | 0.37 (0.28 to 0.5) | 0.1 (0.01 to 0.26) |
|  | YLDs | 23.03 (15.05 to 33.74) | 70.58 (45.82 to 103.43) | 2.06 (1.9 to 2.24) | 0.25 (0.16 to 0.35) | 0.24 (0.16 to 0.34) | 0.01 (-0.03 to 0.06) |
|  | YLLs | 9.94 (5.79 to 14.17) | 38.37 (23.96 to 57.94) | 2.86 (1.24 to 5.21) | 0.09 (0.05 to 0.13) | 0.14 (0.08 to 0.2) | 0.74 (-0.01 to 1.96) |
|  | Deaths | 0.16 (0.09 to 0.23) | 0.68 (0.42 to 1.03) | 3.25 (1.48 to 5.86) | 0 (0 to 0) | 0 (0 to 0) | 0.91 (0.06 to 2.29) |
| Chad | Prevalence | 66.43 (52.13 to 83.81) | 193.68 (148.86 to 248.73) | 1.92 (1.77 to 2.08) | 1.27 (0.99 to 1.57) | 1.26 (0.98 to 1.56) | -0.04 (-0.08 to 0) |
|  | Incidence | 22.04 (18.27 to 26.47) | 55.63 (45.73 to 67.25) | 1.52 (1.41 to 1.65) | 0.46 (0.38 to 0.58) | 0.43 (0.35 to 0.53) | -0.07 (-0.11 to -0.04) |
|  | DALYs | 15.54 (10.6 to 21.4) | 46.78 (32.02 to 66.19) | 2.01 (1.81 to 2.23) | 0.29 (0.2 to 0.4) | 0.3 (0.21 to 0.42) | 0.09 (-0.06 to 0.33) |
|  | YLDs | 14.13 (9.35 to 20.27) | 41.2 (26.81 to 60.49) | 1.92 (1.77 to 2.08) | 0.27 (0.18 to 0.38) | 0.27 (0.18 to 0.38) | -0.04 (-0.08 to 0) |
|  | YLLs | 1.41 (0.77 to 2.55) | 5.58 (3.03 to 9.11) | 2.95 (1.6 to 5.12) | 0.02 (0.01 to 0.04) | 0.03 (0.02 to 0.05) | 0.46 (-0.13 to 1.32) |
|  | Deaths | 0.02 (0.01 to 0.04) | 0.09 (0.05 to 0.14) | 3.1 (1.7 to 5.51) | 0 (0 to 0) | 0 (0 to 0) | 0.48 (-0.12 to 1.42) |
| Côte d’Ivore | Prevalence | 132.93 (101.37 to 169.67) | 308.27 (234.73 to 393.7) | 1.32 (1.2 to 1.46) | 1.23 (0.96 to 1.53) | 1.19 (0.93 to 1.48) | 0 (-0.04 to 0.04) |
|  | Incidence | 34.38 (28.09 to 41.7) | 77.3 (63.21 to 94.07) | 1.25 (1.13 to 1.37) | 0.39 (0.31 to 0.48) | 0.36 (0.29 to 0.44) | -0.08 (-0.11 to -0.04) |
|  | DALYs | 36.32 (26.09 to 49.25) | 86.04 (61.95 to 119.02) | 1.37 (1.09 to 1.7) | 0.32 (0.24 to 0.43) | 0.33 (0.24 to 0.44) | 0.04 (-0.02 to 0.11) |
|  | YLDs | 28.28 (18.72 to 41.87) | 65.59 (42.48 to 95.36) | 1.32 (1.2 to 1.46) | 0.26 (0.17 to 0.37) | 0.25 (0.17 to 0.36) | 0 (-0.04 to 0.04) |
|  | YLLs | 8.05 (4.19 to 11.81) | 20.45 (10.79 to 31.08) | 1.54 (0.48 to 2.85) | 0.06 (0.03 to 0.09) | 0.08 (0.04 to 0.12) | 0.59 (0.04 to 1.53) |
|  | Deaths | 0.12 (0.06 to 0.18) | 0.36 (0.19 to 0.56) | 1.88 (0.65 to 3.35) | 0 (0 to 0) | 0 (0 to 0) | 0.64 (0.01 to 1.68) |
| Gambia | Prevalence | 12.1 (9.36 to 15.37) | 29.47 (22.35 to 37.64) | 1.43 (1.3 to 1.56) | 1.37 (1.08 to 1.7) | 1.3 (1.02 to 1.6) | -0.05 (-0.09 to -0.01) |
|  | Incidence | 3.02 (2.5 to 3.63) | 6.96 (5.66 to 8.47) | 1.31 (1.19 to 1.41) | 0.43 (0.35 to 0.52) | 0.4 (0.32 to 0.48) | -0.07 (-0.1 to -0.04) |
|  | DALYs | 3.04 (2.15 to 4.22) | 7.9 (5.64 to 11.01) | 1.6 (1.32 to 1.94) | 0.34 (0.24 to 0.47) | 0.35 (0.26 to 0.49) | 0.05 (-0.05 to 0.19) |
|  | YLDs | 2.57 (1.68 to 3.75) | 6.27 (4.05 to 9.26) | 1.44 (1.3 to 1.56) | 0.29 (0.19 to 0.41) | 0.28 (0.18 to 0.4) | -0.05 (-0.09 to -0.01) |
|  | YLLs | 0.47 (0.22 to 0.77) | 1.63 (0.96 to 2.58) | 2.51 (1.03 to 5.07) | 0.05 (0.02 to 0.07) | 0.08 (0.05 to 0.12) | 0.66 (-0.02 to 1.98) |
|  | Deaths | 0.01 (0 to 0.01) | 0.03 (0.02 to 0.04) | 3 (1.3 to 6.1) | 0 (0 to 0) | 0 (0 to 0) | 0.71 (0.02 to 2.07) |
| Ghana | Prevalence | 166.32 (126.7 to 208.96) | 399.85 (309.08 to 513.37) | 1.4 (1.28 to 1.53) | 1.22 (0.95 to 1.52) | 1.22 (0.97 to 1.52) | 0 (-0.04 to 0.04) |
|  | Incidence | 44 (36.28 to 52.68) | 94.71 (78.23 to 114.7) | 1.15 (1.03 to 1.25) | 0.39 (0.32 to 0.49) | 0.35 (0.29 to 0.42) | -0.11 (-0.14 to -0.07) |
|  | DALYs | 51.5 (38.53 to 68.44) | 142.11 (104.24 to 189.68) | 1.76 (1.32 to 2.29) | 0.37 (0.28 to 0.49) | 0.44 (0.33 to 0.58) | 0.19 (0.02 to 0.44) |
|  | YLDs | 35.38 (23.09 to 52.65) | 85.06 (55.09 to 124.79) | 1.4 (1.28 to 1.53) | 0.26 (0.17 to 0.37) | 0.26 (0.17 to 0.37) | 0 (-0.04 to 0.04) |
|  | YLLs | 16.12 (11.24 to 21.49) | 57.05 (35.83 to 85.77) | 2.54 (1.21 to 4.52) | 0.11 (0.07 to 0.15) | 0.18 (0.11 to 0.28) | 0.65 (0.06 to 1.55) |
|  | Deaths | 0.27 (0.18 to 0.36) | 1.08 (0.65 to 1.68) | 3.05 (1.54 to 5.31) | 0 (0 to 0) | 0 (0 to 0.01) | 0.74 (0.09 to 1.71) |
| Guinea | Prevalence | 63.7 (49.77 to 79.99) | 141.46 (107.64 to 181.13) | 1.22 (1.1 to 1.34) | 1.2 (0.93 to 1.5) | 1.16 (0.9 to 1.45) | -0.03 (-0.07 to 0.01) |
|  | Incidence | 21.14 (17.45 to 25.34) | 39.73 (32.75 to 47.84) | 0.88 (0.79 to 0.97) | 0.42 (0.34 to 0.52) | 0.39 (0.32 to 0.48) | -0.08 (-0.11 to -0.04) |
|  | DALYs | 16.6 (11.95 to 22.55) | 37.76 (26.86 to 52.45) | 1.27 (1.04 to 1.54) | 0.3 (0.21 to 0.41) | 0.31 (0.22 to 0.42) | 0.04 (-0.05 to 0.15) |
|  | YLDs | 13.55 (8.94 to 19.79) | 30.1 (19.44 to 43.84) | 1.22 (1.1 to 1.34) | 0.25 (0.17 to 0.36) | 0.25 (0.16 to 0.35) | -0.03 (-0.07 to 0.01) |
|  | YLLs | 3.05 (1.92 to 4.98) | 7.66 (4.09 to 11.87) | 1.51 (0.6 to 3.11) | 0.04 (0.03 to 0.07) | 0.06 (0.03 to 0.09) | 0.46 (-0.08 to 1.28) |
|  | Deaths | 0.05 (0.03 to 0.07) | 0.13 (0.07 to 0.2) | 1.8 (0.78 to 3.45) | 0 (0 to 0) | 0 (0 to 0) | 0.55 (-0.04 to 1.49) |
| Guinea-Bissau | Prevalence | 10.51 (8.07 to 13.49) | 21.62 (16.5 to 27.88) | 1.06 (0.97 to 1.15) | 1.16 (0.91 to 1.44) | 1.12 (0.88 to 1.39) | -0.04 (-0.07 to 0) |
|  | Incidence | 3.31 (2.73 to 3.99) | 6.2 (5.07 to 7.62) | 0.87 (0.8 to 0.94) | 0.45 (0.36 to 0.55) | 0.41 (0.34 to 0.51) | -0.08 (-0.11 to -0.05) |
|  | DALYs | 2.84 (2.07 to 3.87) | 6.03 (4.36 to 8.41) | 1.12 (0.88 to 1.38) | 0.31 (0.23 to 0.42) | 0.32 (0.24 to 0.43) | 0.03 (-0.08 to 0.17) |
|  | YLDs | 2.24 (1.44 to 3.26) | 4.6 (2.95 to 6.74) | 1.06 (0.97 to 1.15) | 0.25 (0.16 to 0.35) | 0.24 (0.16 to 0.34) | -0.04 (-0.07 to 0) |
|  | YLLs | 0.6 (0.37 to 0.87) | 1.43 (0.92 to 2.04) | 1.37 (0.42 to 2.71) | 0.06 (0.04 to 0.09) | 0.08 (0.05 to 0.12) | 0.3 (-0.21 to 0.97) |
|  | Deaths | 0.01 (0.01 to 0.01) | 0.03 (0.02 to 0.04) | 1.64 (0.58 to 3.01) | 0 (0 to 0) | 0 (0 to 0) | 0.34 (-0.19 to 1.13) |
| Liberia | Prevalence | 24.79 (18.97 to 31.6) | 54.95 (41.34 to 70.47) | 1.22 (1.09 to 1.33) | 1.13 (0.89 to 1.41) | 1.07 (0.83 to 1.34) | -0.06 (-0.09 to -0.02) |
|  | Incidence | 7.66 (6.35 to 9.16) | 15.56 (12.69 to 19) | 1.03 (0.9 to 1.15) | 0.4 (0.32 to 0.49) | 0.38 (0.31 to 0.46) | -0.06 (-0.09 to -0.02) |
|  | DALYs | 7 (5.14 to 9.49) | 14.66 (10.34 to 19.97) | 1.09 (0.84 to 1.36) | 0.3 (0.22 to 0.4) | 0.29 (0.21 to 0.38) | -0.05 (-0.13 to 0.07) |
|  | YLDs | 5.27 (3.42 to 7.74) | 11.69 (7.46 to 17.05) | 1.22 (1.09 to 1.33) | 0.24 (0.16 to 0.34) | 0.23 (0.15 to 0.32) | -0.06 (-0.09 to -0.02) |
|  | YLLs | 1.73 (1.07 to 2.48) | 2.97 (1.67 to 4.57) | 0.71 (0.01 to 1.77) | 0.06 (0.03 to 0.08) | 0.06 (0.03 to 0.09) | -0.01 (-0.4 to 0.5) |
|  | Deaths | 0.03 (0.01 to 0.04) | 0.05 (0.03 to 0.08) | 1.08 (0.3 to 2.2) | 0 (0 to 0) | 0 (0 to 0) | 0.08 (-0.33 to 0.62) |
| Mali | Prevalence | 132.39 (106.35 to 160.36) | 363.98 (293.71 to 442.9) | 1.75 (1.61 to 1.9) | 1.96 (1.61 to 2.34) | 2.03 (1.68 to 2.39) | 0.03 (-0.01 to 0.09) |
|  | Incidence | 67.74 (60.38 to 75.19) | 157.05 (142.09 to 172.67) | 1.32 (1.22 to 1.42) | 1.16 (1.04 to 1.29) | 1.13 (1.03 to 1.23) | -0.03 (-0.08 to 0.02) |
|  | DALYs | 31.4 (22.56 to 42.38) | 87.15 (60.98 to 118.01) | 1.78 (1.61 to 1.97) | 0.45 (0.32 to 0.6) | 0.48 (0.34 to 0.63) | 0.06 (0 to 0.13) |
|  | YLDs | 28.15 (19.25 to 39.31) | 77.4 (51.89 to 109.01) | 1.75 (1.61 to 1.9) | 0.42 (0.29 to 0.56) | 0.43 (0.3 to 0.58) | 0.03 (-0.01 to 0.09) |
|  | YLLs | 3.25 (1.87 to 5.47) | 9.75 (5.24 to 15.42) | 2 (1.11 to 3.38) | 0.03 (0.02 to 0.06) | 0.05 (0.03 to 0.07) | 0.32 (-0.08 to 0.98) |
|  | Deaths | 0.05 (0.03 to 0.09) | 0.16 (0.09 to 0.26) | 2.17 (1.22 to 3.65) | 0 (0 to 0) | 0 (0 to 0) | 0.38 (-0.06 to 1.1) |
| Mauritania | Prevalence | 29.42 (23.02 to 36.87) | 63.39 (49.42 to 79.35) | 1.16 (1.06 to 1.25) | 1.58 (1.25 to 1.96) | 1.54 (1.22 to 1.91) | -0.02 (-0.06 to 0.02) |
|  | Incidence | 7.26 (6.06 to 8.64) | 14.14 (11.69 to 17.08) | 0.95 (0.87 to 1.02) | 0.45 (0.37 to 0.56) | 0.41 (0.34 to 0.51) | -0.09 (-0.11 to -0.06) |
|  | DALYs | 7.85 (5.73 to 10.88) | 17.28 (12.44 to 23.78) | 1.2 (1 to 1.44) | 0.42 (0.3 to 0.56) | 0.43 (0.32 to 0.58) | 0.04 (-0.07 to 0.16) |
|  | YLDs | 6.26 (4.14 to 8.96) | 13.48 (8.98 to 19.59) | 1.16 (1.06 to 1.25) | 0.34 (0.22 to 0.47) | 0.33 (0.22 to 0.47) | -0.02 (-0.06 to 0.02) |
|  | YLLs | 1.59 (1.11 to 2.14) | 3.79 (2.43 to 5.65) | 1.38 (0.53 to 2.61) | 0.08 (0.06 to 0.11) | 0.1 (0.06 to 0.16) | 0.28 (-0.18 to 1.02) |
|  | Deaths | 0.03 (0.02 to 0.04) | 0.07 (0.04 to 0.11) | 1.67 (0.71 to 3.14) | 0 (0 to 0) | 0 (0 to 0) | 0.32 (-0.17 to 1.16) |
| Niger | Prevalence | 89.65 (69.68 to 113.11) | 272.74 (208.96 to 345.39) | 2.04 (1.9 to 2.2) | 1.31 (1.02 to 1.62) | 1.28 (1.01 to 1.58) | -0.03 (-0.06 to 0.01) |
|  | Incidence | 28.87 (23.65 to 34.69) | 82.67 (68.68 to 98.88) | 1.86 (1.74 to 1.98) | 0.48 (0.39 to 0.59) | 0.46 (0.37 to 0.56) | -0.05 (-0.07 to -0.01) |
|  | DALYs | 20.86 (14.1 to 29.11) | 60.94 (40.09 to 86.3) | 1.92 (1.73 to 2.11) | 0.3 (0.2 to 0.41) | 0.28 (0.19 to 0.39) | -0.04 (-0.08 to 0) |
|  | YLDs | 19.07 (12.37 to 27.47) | 58.01 (37.45 to 84.07) | 2.04 (1.9 to 2.2) | 0.28 (0.19 to 0.4) | 0.27 (0.18 to 0.38) | -0.03 (-0.06 to 0.01) |
|  | YLLs | 1.79 (0.92 to 3.58) | 2.93 (1.1 to 6.47) | 0.63 (-0.09 to 1.7) | 0.02 (0.01 to 0.03) | 0.01 (0.01 to 0.03) | -0.23 (-0.52 to 0.2) |
|  | Deaths | 0.03 (0.01 to 0.05) | 0.05 (0.02 to 0.11) | 0.92 (0.13 to 2.03) | 0 (0 to 0) | 0 (0 to 0) | -0.12 (-0.45 to 0.4) |
| Nigeria | Prevalence | 1081.46 (807.25 to 1388.86) | 2640.4 (1940.6 to 3420.65) | 1.44 (1.38 to 1.51) | 1.31 (0.99 to 1.67) | 1.23 (0.93 to 1.58) | -0.06 (-0.08 to -0.05) |
|  | Incidence | 281.97 (230.91 to 344.4) | 600.14 (488.97 to 735.94) | 1.13 (1.07 to 1.19) | 0.38 (0.31 to 0.48) | 0.33 (0.27 to 0.41) | -0.15 (-0.17 to -0.13) |
|  | DALYs | 292.26 (210.23 to 402.37) | 813.12 (587.57 to 1102.62) | 1.78 (1.54 to 2.22) | 0.34 (0.25 to 0.47) | 0.38 (0.28 to 0.52) | 0.13 (0.01 to 0.33) |
|  | YLDs | 230.1 (145.04 to 336.15) | 561.77 (351.93 to 830.33) | 1.44 (1.38 to 1.51) | 0.28 (0.18 to 0.4) | 0.26 (0.17 to 0.38) | -0.06 (-0.08 to -0.05) |
|  | YLLs | 62.16 (43.11 to 80.47) | 251.34 (167.83 to 371.66) | 3.04 (1.92 to 4.56) | 0.06 (0.04 to 0.08) | 0.12 (0.08 to 0.19) | 0.98 (0.34 to 1.92) |
|  | Deaths | 0.97 (0.63 to 1.29) | 4.5 (2.96 to 6.84) | 3.62 (2.17 to 5.62) | 0 (0 to 0) | 0 (0 to 0) | 1.14 (0.38 to 2.35) |
| Sao Tome and Principe | Prevalence | 1.32 (1.02 to 1.68) | 2.76 (2.2 to 3.38) | 1.08 (0.95 to 1.23) | 1.22 (0.96 to 1.5) | 1.41 (1.14 to 1.69) | 0.16 (0.1 to 0.23) |
|  | Incidence | 0.38 (0.32 to 0.44) | 0.85 (0.73 to 0.96) | 1.2 (1.06 to 1.36) | 0.41 (0.35 to 0.48) | 0.54 (0.48 to 0.61) | 0.33 (0.24 to 0.43) |
|  | DALYs | 0.36 (0.26 to 0.5) | 0.9 (0.65 to 1.18) | 1.47 (1.05 to 1.99) | 0.33 (0.24 to 0.44) | 0.46 (0.34 to 0.59) | 0.39 (0.17 to 0.68) |
|  | YLDs | 0.28 (0.19 to 0.41) | 0.59 (0.39 to 0.83) | 1.08 (0.95 to 1.23) | 0.26 (0.17 to 0.37) | 0.3 (0.2 to 0.41) | 0.16 (0.1 to 0.23) |
|  | YLLs | 0.08 (0.04 to 0.12) | 0.31 (0.2 to 0.49) | 2.78 (0.98 to 5.49) | 0.07 (0.04 to 0.1) | 0.16 (0.1 to 0.25) | 1.26 (0.25 to 2.85) |
|  | Deaths | 0 (0 to 0) | 0.01 (0 to 0.01) | 3.62 (1.47 to 6.83) | 0 (0 to 0) | 0 (0 to 0.01) | 1.51 (0.37 to 3.25) |
| Senegal | Prevalence | 93.94 (73 to 118.25) | 203.47 (157.94 to 256.86) | 1.17 (1.05 to 1.27) | 1.39 (1.08 to 1.73) | 1.36 (1.08 to 1.69) | -0.02 (-0.06 to 0.02) |
|  | Incidence | 24.41 (20.16 to 29.23) | 48.08 (39.31 to 58.03) | 0.97 (0.86 to 1.06) | 0.42 (0.34 to 0.52) | 0.39 (0.31 to 0.48) | -0.08 (-0.1 to -0.05) |
|  | DALYs | 24.63 (17.43 to 34.03) | 55.38 (39.04 to 77.56) | 1.25 (0.96 to 1.64) | 0.35 (0.25 to 0.48) | 0.38 (0.27 to 0.52) | 0.06 (-0.06 to 0.24) |
|  | YLDs | 19.98 (13.14 to 29.07) | 43.3 (28.2 to 63.46) | 1.17 (1.05 to 1.27) | 0.3 (0.2 to 0.42) | 0.29 (0.19 to 0.41) | -0.02 (-0.06 to 0.02) |
|  | YLLs | 4.65 (2.64 to 7.35) | 12.09 (6.5 to 20.68) | 1.6 (0.42 to 3.46) | 0.06 (0.03 to 0.09) | 0.09 (0.05 to 0.15) | 0.47 (-0.17 to 1.47) |
|  | Deaths | 0.07 (0.04 to 0.11) | 0.22 (0.12 to 0.38) | 2.07 (0.69 to 4.19) | 0 (0 to 0) | 0 (0 to 0) | 0.56 (-0.14 to 1.6) |
| Sierra Leona | Prevalence | 42.77 (32.9 to 54.87) | 91.08 (68.91 to 116.59) | 1.13 (1.03 to 1.24) | 1.15 (0.9 to 1.43) | 1.1 (0.86 to 1.36) | -0.04 (-0.08 to 0) |
|  | Incidence | 13.8 (11.39 to 16.55) | 26.26 (21.61 to 31.88) | 0.9 (0.82 to 0.98) | 0.42 (0.34 to 0.52) | 0.39 (0.32 to 0.48) | -0.06 (-0.09 to -0.03) |
|  | DALYs | 11.53 (8.38 to 15.69) | 24.33 (17.34 to 33.72) | 1.11 (0.89 to 1.33) | 0.29 (0.21 to 0.39) | 0.29 (0.21 to 0.4) | 0.01 (-0.08 to 0.11) |
|  | YLDs | 9.1 (5.93 to 13.24) | 19.38 (12.39 to 28.29) | 1.13 (1.03 to 1.24) | 0.24 (0.16 to 0.35) | 0.23 (0.15 to 0.33) | -0.04 (-0.08 to 0) |
|  | YLLs | 2.43 (1.39 to 3.39) | 4.95 (2.72 to 7.41) | 1.03 (0.25 to 2.11) | 0.05 (0.03 to 0.07) | 0.06 (0.03 to 0.09) | 0.25 (-0.21 to 0.87) |
|  | Deaths | 0.04 (0.02 to 0.05) | 0.08 (0.05 to 0.12) | 1.37 (0.48 to 2.53) | 0 (0 to 0) | 0 (0 to 0) | 0.35 (-0.13 to 1.1) |
| Togo | Prevalence | 38.38 (29.27 to 49.31) | 90.49 (69.77 to 115.8) | 1.36 (1.23 to 1.5) | 1.18 (0.92 to 1.47) | 1.14 (0.89 to 1.42) | -0.03 (-0.07 to 0) |
|  | Incidence | 10.56 (8.64 to 12.78) | 24.71 (20.09 to 30.31) | 1.34 (1.19 to 1.5) | 0.41 (0.32 to 0.5) | 0.38 (0.31 to 0.47) | -0.07 (-0.1 to -0.04) |
|  | DALYs | 10.31 (7.41 to 14.28) | 24.8 (17.67 to 33.49) | 1.41 (1.18 to 1.69) | 0.31 (0.22 to 0.42) | 0.31 (0.23 to 0.42) | 0.01 (-0.08 to 0.12) |
|  | YLDs | 8.16 (5.3 to 12.03) | 19.25 (12.39 to 28.05) | 1.36 (1.23 to 1.5) | 0.25 (0.17 to 0.36) | 0.24 (0.16 to 0.35) | -0.03 (-0.07 to 0) |
|  | YLLs | 2.14 (1.27 to 3.02) | 5.55 (3.28 to 8.37) | 1.59 (0.68 to 2.83) | 0.06 (0.04 to 0.08) | 0.07 (0.04 to 0.11) | 0.21 (-0.23 to 0.76) |
|  | Deaths | 0.03 (0.02 to 0.05) | 0.1 (0.06 to 0.16) | 2.06 (0.95 to 3.55) | 0 (0 to 0) | 0 (0 to 0) | 0.25 (-0.22 to 0.88) |
| Central Sub Sahara |  |  |  |  |  |  |  |
| Angola | Prevalence | 121.45 (94.8 to 152.66) | 387.8 (298.4 to 495.48) | 2.19 (2.05 to 2.36) | 1.18 (0.92 to 1.49) | 1.19 (0.91 to 1.51) | -0.01 (-0.05 to 0.03) |
|  | Incidence | 35.97 (29.38 to 43.48) | 100.92 (82.47 to 123.41) | 1.81 (1.71 to 1.93) | 0.35 (0.29 to 0.42) | 0.31 (0.25 to 0.38) | -0.12 (-0.15 to -0.08) |
|  | DALYs | 31.26 (22.6 to 43.94) | 88.85 (59.81 to 129.11) | 1.84 (1.49 to 2.2) | 0.3 (0.22 to 0.43) | 0.27 (0.18 to 0.39) | -0.09 (-0.17 to 0.02) |
|  | YLDs | 25.84 (16.83 to 37.82) | 82.51 (54.48 to 121.74) | 2.19 (2.05 to 2.36) | 0.25 (0.16 to 0.37) | 0.25 (0.17 to 0.37) | -0.01 (-0.05 to 0.03) |
|  | YLLs | 5.42 (3.01 to 8.4) | 6.34 (3.24 to 15.71) | 0.17 (-0.48 to 1.87) | 0.05 (0.03 to 0.08) | 0.02 (0.01 to 0.05) | -0.51 (-0.77 to 0.18) |
|  | Deaths | 0.09 (0.06 to 0.14) | 0.13 (0.06 to 0.34) | 0.36 (-0.36 to 2.22) | 0 (0 to 0) | 0 (0 to 0) | -0.51 (-0.76 to 0.27) |
| Central African Republic | Prevalence | 25.82 (19.64 to 32.98) | 50.21 (37.68 to 64.67) | 0.94 (0.86 to 1.04) | 0.95 (0.72 to 1.21) | 0.92 (0.69 to 1.18) | -0.07 (-0.1 to -0.02) |
|  | Incidence | 9.88 (8.08 to 12.15) | 18.87 (15.16 to 23.41) | 0.91 (0.82 to 1) | 0.36 (0.3 to 0.45) | 0.34 (0.28 to 0.43) | -0.05 (-0.09 to -0.01) |
|  | DALYs | 6.73 (4.83 to 9.38) | 11.25 (7.33 to 16.23) | 0.67 (0.46 to 0.83) | 0.25 (0.18 to 0.34) | 0.21 (0.13 to 0.3) | -0.17 (-0.25 to -0.1) |
|  | YLDs | 5.49 (3.57 to 7.97) | 10.68 (6.83 to 15.64) | 0.94 (0.86 to 1.04) | 0.2 (0.13 to 0.29) | 0.19 (0.12 to 0.29) | -0.07 (-0.1 to -0.02) |
|  | YLLs | 1.24 (0.77 to 1.85) | 0.57 (0.29 to 1.35) | -0.54 (-0.77 to -0.01) | 0.05 (0.03 to 0.07) | 0.01 (0.01 to 0.02) | -0.71 (-0.84 to -0.35) |
|  | Deaths | 0.02 (0.01 to 0.03) | 0.01 (0.01 to 0.03) | -0.48 (-0.72 to 0.19) | 0 (0 to 0) | 0 (0 to 0) | -0.71 (-0.84 to -0.3) |
| Congo | Prevalence | 24.61 (18.89 to 31.42) | 56.79 (43.86 to 73.29) | 1.31 (1.18 to 1.44) | 1.02 (0.79 to 1.31) | 1.05 (0.81 to 1.36) | -0.03 (-0.06 to 0.01) |
|  | Incidence | 6.79 (5.52 to 8.29) | 15.66 (12.4 to 19.68) | 1.31 (1.13 to 1.46) | 0.28 (0.23 to 0.35) | 0.29 (0.23 to 0.36) | -0.08 (-0.11 to -0.04) |
|  | DALYs | 7.79 (5.45 to 10.61) | 14.26 (9.84 to 20.2) | 0.83 (0.49 to 1.17) | 0.32 (0.23 to 0.44) | 0.26 (0.18 to 0.37) | -0.21 (-0.34 to -0.08) |
|  | YLDs | 5.24 (3.43 to 7.66) | 12.08 (7.86 to 17.67) | 1.31 (1.18 to 1.44) | 0.22 (0.14 to 0.32) | 0.22 (0.15 to 0.33) | -0.03 (-0.06 to 0.01) |
|  | YLLs | 2.55 (1.22 to 3.97) | 2.18 (1.11 to 3.88) | -0.15 (-0.51 to 0.58) | 0.11 (0.05 to 0.17) | 0.04 (0.02 to 0.07) | -0.57 (-0.75 to -0.21) |
|  | Deaths | 0.05 (0.03 to 0.07) | 0.05 (0.03 to 0.1) | 0.03 (-0.4 to 0.89) | 0 (0 to 0) | 0 (0 to 0) | -0.57 (-0.75 to -0.21) |
| Democratic Republic of the Congo | Prevalence | 370.22 (283.16 to 470.44) | 847.46 (645.11 to 1093.77) | 1.29 (1.18 to 1.38) | 0.97 (0.74 to 1.23) | 0.94 (0.72 to 1.22) | -0.08 (-0.11 to -0.05) |
|  | Incidence | 118.28 (97.14 to 142.23) | 268.79 (219.6 to 324) | 1.27 (1.17 to 1.38) | 0.31 (0.25 to 0.37) | 0.3 (0.24 to 0.36) | -0.04 (-0.07 to 0) |
|  | DALYs | 103 (74.18 to 143.6) | 192.15 (127.94 to 276.75) | 0.87 (0.58 to 1.13) | 0.27 (0.19 to 0.38) | 0.21 (0.14 to 0.31) | -0.2 (-0.29 to -0.1) |
|  | YLDs | 78.75 (51.45 to 116.08) | 180.26 (116.75 to 261.89) | 1.29 (1.18 to 1.38) | 0.21 (0.13 to 0.3) | 0.2 (0.13 to 0.29) | -0.08 (-0.11 to -0.05) |
|  | YLLs | 24.25 (14.36 to 37.97) | 11.89 (6.37 to 29.4) | -0.51 (-0.77 to 0.2) | 0.06 (0.04 to 0.1) | 0.01 (0.01 to 0.03) | -0.69 (-0.84 to -0.29) |
|  | Deaths | 0.42 (0.26 to 0.63) | 0.27 (0.14 to 0.71) | -0.37 (-0.68 to 0.45) | 0 (0 to 0) | 0 (0 to 0) | -0.67 (-0.82 to -0.2) |
| Equatorial Guinea | Prevalence | 4.07 (3.13 to 5.17) | 17.26 (13.15 to 22.52) | 3.24 (2.98 to 3.5) | 0.96 (0.74 to 1.22) | 1.14 (0.87 to 1.49) | 0.1 (0.06 to 0.15) |
|  | Incidence | 1.32 (1.08 to 1.59) | 3.45 (2.81 to 4.23) | 1.62 (1.42 to 1.82) | 0.31 (0.26 to 0.38) | 0.23 (0.19 to 0.28) | -0.19 (-0.23 to -0.16) |
|  | DALYs | 1.09 (0.79 to 1.52) | 4.26 (2.91 to 6.09) | 2.91 (2.43 to 3.4) | 0.26 (0.19 to 0.36) | 0.28 (0.19 to 0.4) | 0.11 (-0.04 to 0.32) |
|  | YLDs | 0.87 (0.57 to 1.28) | 3.67 (2.36 to 5.48) | 3.24 (2.98 to 3.5) | 0.2 (0.13 to 0.3) | 0.24 (0.16 to 0.36) | 0.1 (0.06 to 0.15) |
|  | YLLs | 0.22 (0.14 to 0.33) | 0.58 (0.28 to 1.06) | 1.61 (0.22 to 3.85) | 0.05 (0.03 to 0.08) | 0.04 (0.02 to 0.07) | 0.15 (-0.48 to 1.27) |
|  | Deaths | 0 (0 to 0.01) | 0.01 (0.01 to 0.02) | 2 (0.36 to 4.84) | 0 (0 to 0) | 0 (0 to 0) | 0.16 (-0.52 to 1.44) |
| Gabon | Prevalence | 11.38 (9 to 14.36) | 21.02 (16.22 to 26.44) | 0.85 (0.75 to 0.95) | 1.16 (0.91 to 1.46) | 1.16 (0.89 to 1.46) | -0.05 (-0.08 to 0) |
|  | Incidence | 2.79 (2.28 to 3.37) | 4.9 (3.94 to 6) | 0.76 (0.66 to 0.84) | 0.28 (0.23 to 0.34) | 0.27 (0.22 to 0.33) | -0.08 (-0.11 to -0.05) |
|  | DALYs | 3.53 (2.55 to 4.8) | 5.3 (3.72 to 7.56) | 0.5 (0.27 to 0.74) | 0.36 (0.26 to 0.49) | 0.29 (0.2 to 0.42) | -0.2 (-0.32 to -0.06) |
|  | YLDs | 2.42 (1.59 to 3.52) | 4.47 (2.94 to 6.5) | 0.85 (0.75 to 0.95) | 0.25 (0.16 to 0.36) | 0.25 (0.16 to 0.36) | -0.05 (-0.08 to 0) |
|  | YLLs | 1.11 (0.68 to 1.65) | 0.83 (0.48 to 1.68) | -0.25 (-0.58 to 0.45) | 0.11 (0.07 to 0.17) | 0.05 (0.03 to 0.09) | -0.55 (-0.75 to -0.13) |
|  | Deaths | 0.03 (0.02 to 0.04) | 0.02 (0.01 to 0.04) | -0.18 (-0.55 to 0.62) | 0 (0 to 0) | 0 (0 to 0) | -0.55 (-0.77 to -0.09) |
| Eastern Sub-Sahara |  |  |  |  |  |  |  |
| Burundi | Prevalence | 53.04 (40.76 to 67.63) | 123.23 (93.59 to 157.62) | 1.32 (1.22 to 1.44) | 1.1 (0.86 to 1.38) | 1.04 (0.81 to 1.31) | -0.05 (-0.09 to -0.01) |
|  | Incidence | 18.33 (15 to 22.2) | 41.17 (33.45 to 50.54) | 1.25 (1.13 to 1.35) | 0.47 (0.37 to 0.59) | 0.46 (0.36 to 0.57) | -0.02 (-0.06 to 0.02) |
|  | DALYs | 13.16 (9.3 to 18.47) | 27.04 (17.91 to 39.55) | 1.05 (0.86 to 1.21) | 0.27 (0.19 to 0.37) | 0.23 (0.16 to 0.32) | -0.14 (-0.24 to -0.08) |
|  | YLDs | 11.28 (7.31 to 16.51) | 26.22 (17.19 to 38.66) | 1.32 (1.22 to 1.44) | 0.23 (0.16 to 0.34) | 0.22 (0.15 to 0.32) | -0.05 (-0.09 to -0.01) |
|  | YLLs | 1.88 (1.1 to 2.9) | 0.83 (0.29 to 1.98) | -0.56 (-0.81 to 0.07) | 0.03 (0.02 to 0.07) | 0.01 (0 to 0.02) | -0.77 (-0.89 to -0.42) |
|  | Deaths | 0.03 (0.02 to 0.06) | 0.02 (0.01 to 0.04) | -0.52 (-0.78 to 0.22) | 0 (0 to 0) | 0 (0 to 0) | -0.77 (-0.89 to -0.36) |
| Comoros | Prevalence | 5.92 (4.59 to 7.48) | 10.04 (7.76 to 12.7) | 0.7 (0.58 to 0.83) | 1.46 (1.15 to 1.82) | 1.39 (1.09 to 1.73) | -0.05 (-0.09 to -0.01) |
|  | Incidence | 1.57 (1.28 to 1.91) | 2.83 (2.27 to 3.5) | 0.8 (0.65 to 0.93) | 0.49 (0.39 to 0.6) | 0.45 (0.36 to 0.56) | -0.06 (-0.1 to -0.03) |
|  | DALYs | 1.46 (1.04 to 2.06) | 2.24 (1.51 to 3.19) | 0.53 (0.35 to 0.68) | 0.35 (0.25 to 0.48) | 0.31 (0.21 to 0.44) | -0.11 (-0.18 to -0.05) |
|  | YLDs | 1.26 (0.83 to 1.84) | 2.14 (1.4 to 3.1) | 0.7 (0.58 to 0.83) | 0.31 (0.21 to 0.44) | 0.3 (0.2 to 0.42) | -0.05 (-0.09 to -0.01) |
|  | YLLs | 0.2 (0.1 to 0.34) | 0.11 (0.06 to 0.18) | -0.47 (-0.69 to 0) | 0.04 (0.02 to 0.07) | 0.02 (0.01 to 0.03) | -0.6 (-0.76 to -0.29) |
|  | Deaths | 0 (0 to 0.01) | 0 (0 to 0) | -0.27 (-0.56 to 0.33) | 0 (0 to 0) | 0 (0 to 0) | -0.59 (-0.76 to -0.23) |
| Djibouti | Prevalence | 5.52 (4.24 to 7) | 17.35 (13.53 to 22.09) | 2.14 (1.94 to 2.36) | 1.49 (1.17 to 1.84) | 1.43 (1.13 to 1.78) | -0.04 (-0.07 to 0) |
|  | Incidence | 1.31 (1.06 to 1.62) | 4.5 (3.6 to 5.62) | 2.44 (2.22 to 2.67) | 0.48 (0.38 to 0.6) | 0.45 (0.36 to 0.56) | -0.07 (-0.1 to -0.03) |
|  | DALYs | 1.36 (0.96 to 1.92) | 3.86 (2.61 to 5.58) | 1.85 (1.52 to 2.13) | 0.37 (0.26 to 0.5) | 0.32 (0.22 to 0.45) | -0.13 (-0.2 to -0.06) |
|  | YLDs | 1.18 (0.77 to 1.73) | 3.69 (2.42 to 5.36) | 2.14 (1.94 to 2.36) | 0.32 (0.21 to 0.45) | 0.3 (0.2 to 0.43) | -0.04 (-0.07 to 0) |
|  | YLLs | 0.18 (0.1 to 0.3) | 0.17 (0.07 to 0.47) | -0.05 (-0.58 to 1.43) | 0.05 (0.03 to 0.08) | 0.02 (0.01 to 0.05) | -0.67 (-0.84 to -0.21) |
|  | Deaths | 0 (0 to 0) | 0 (0 to 0.01) | 0.24 (-0.43 to 2.06) | 0 (0 to 0) | 0 (0 to 0) | -0.67 (-0.84 to -0.18) |
| Eritrea | Prevalence | 40.81 (31.36 to 52.19) | 82.87 (63.42 to 106.13) | 1.03 (0.91 to 1.14) | 1.36 (1.07 to 1.7) | 1.34 (1.05 to 1.65) | -0.02 (-0.05 to 0.02) |
|  | Incidence | 12.74 (10.35 to 15.74) | 24.44 (19.86 to 30.15) | 0.92 (0.82 to 1.02) | 0.56 (0.45 to 0.71) | 0.51 (0.41 to 0.64) | -0.1 (-0.13 to -0.06) |
|  | DALYs | 9.64 (6.74 to 13.7) | 18.59 (12.23 to 26.47) | 0.93 (0.78 to 1.08) | 0.32 (0.23 to 0.45) | 0.3 (0.21 to 0.42) | -0.06 (-0.12 to 0.01) |
|  | YLDs | 8.68 (5.64 to 12.75) | 17.63 (11.37 to 25.43) | 1.03 (0.91 to 1.14) | 0.29 (0.19 to 0.41) | 0.28 (0.19 to 0.4) | -0.02 (-0.05 to 0.02) |
|  | YLLs | 0.96 (0.6 to 1.6) | 0.96 (0.44 to 2.45) | 0 (-0.5 to 1.02) | 0.03 (0.02 to 0.06) | 0.02 (0.01 to 0.05) | -0.45 (-0.68 to 0.14) |
|  | Deaths | 0.02 (0.01 to 0.03) | 0.02 (0.01 to 0.05) | 0.16 (-0.36 to 1.34) | 0 (0 to 0) | 0 (0 to 0) | -0.47 (-0.69 to 0.16) |
| Ethiopia | Prevalence | 549.43 (407.55 to 714.18) | 1238.1 (909.91 to 1630.3) | 1.25 (1.17 to 1.33) | 1.24 (0.94 to 1.56) | 1.2 (0.91 to 1.53) | -0.03 (-0.05 to 0) |
|  | Incidence | 184.83 (149.9 to 229.52) | 343.44 (276.53 to 433.41) | 0.86 (0.79 to 0.93) | 0.52 (0.41 to 0.66) | 0.44 (0.35 to 0.56) | -0.15 (-0.17 to -0.14) |
|  | DALYs | 130.63 (87.95 to 187.16) | 272.5 (171.17 to 400.17) | 1.09 (0.93 to 1.22) | 0.29 (0.2 to 0.4) | 0.27 (0.18 to 0.38) | -0.07 (-0.13 to -0.02) |
|  | YLDs | 116.89 (73.53 to 171.6) | 263.43 (164.52 to 387.94) | 1.25 (1.17 to 1.33) | 0.26 (0.17 to 0.38) | 0.26 (0.17 to 0.37) | -0.03 (-0.05 to 0) |
|  | YLLs | 13.74 (9.41 to 19.84) | 9.07 (4.41 to 20.86) | -0.34 (-0.66 to 0.36) | 0.03 (0.02 to 0.05) | 0.01 (0.01 to 0.03) | -0.56 (-0.74 to -0.16) |
|  | Deaths | 0.24 (0.16 to 0.4) | 0.19 (0.09 to 0.48) | -0.19 (-0.53 to 0.64) | 0 (0 to 0) | 0 (0 to 0) | -0.54 (-0.71 to -0.04) |
| Kenya | Prevalence | 270.64 (199 to 348.63) | 591.04 (438.96 to 767.55) | 1.18 (1.09 to 1.27) | 1.31 (0.99 to 1.67) | 1.2 (0.91 to 1.53) | -0.08 (-0.1 to -0.06) |
|  | Incidence | 65.79 (53.9 to 81.31) | 131.77 (104.26 to 166.11) | 1 (0.88 to 1.11) | 0.42 (0.33 to 0.54) | 0.35 (0.28 to 0.44) | -0.18 (-0.19 to -0.16) |
|  | DALYs | 66.95 (46.25 to 96.52) | 134.66 (88.06 to 194.56) | 1.01 (0.85 to 1.12) | 0.32 (0.23 to 0.45) | 0.28 (0.19 to 0.39) | -0.13 (-0.18 to -0.08) |
|  | YLDs | 57.58 (36.64 to 85.53) | 125.76 (79.93 to 184.92) | 1.18 (1.09 to 1.27) | 0.28 (0.18 to 0.4) | 0.26 (0.17 to 0.37) | -0.08 (-0.1 to -0.06) |
|  | YLLs | 9.37 (6.95 to 12.36) | 8.9 (6.37 to 15.43) | -0.05 (-0.32 to 0.44) | 0.04 (0.03 to 0.06) | 0.02 (0.02 to 0.04) | -0.42 (-0.6 to -0.08) |
|  | Deaths | 0.16 (0.12 to 0.22) | 0.21 (0.15 to 0.37) | 0.3 (-0.09 to 1.01) | 0 (0 to 0) | 0 (0 to 0) | -0.39 (-0.62 to 0.06) |
| Madagascar | Prevalence | 159.58 (125.12 to 199.66) | 382.95 (298.42 to 484.21) | 1.4 (1.29 to 1.51) | 1.54 (1.21 to 1.9) | 1.47 (1.16 to 1.83) | -0.05 (-0.08 to 0) |
|  | Incidence | 48 (39.62 to 58.41) | 106.23 (87.13 to 130.47) | 1.21 (1.12 to 1.31) | 0.58 (0.46 to 0.73) | 0.53 (0.43 to 0.67) | -0.08 (-0.11 to -0.05) |
|  | DALYs | 40.02 (28.4 to 55.14) | 84.31 (55.08 to 120.72) | 1.11 (0.86 to 1.29) | 0.38 (0.27 to 0.51) | 0.33 (0.22 to 0.46) | -0.13 (-0.2 to -0.08) |
|  | YLDs | 33.94 (22.22 to 49.37) | 81.46 (52.84 to 117.24) | 1.4 (1.29 to 1.51) | 0.33 (0.22 to 0.46) | 0.31 (0.21 to 0.44) | -0.05 (-0.08 to 0) |
|  | YLLs | 6.08 (3.52 to 9.47) | 2.85 (1.63 to 5.78) | -0.53 (-0.76 to -0.03) | 0.05 (0.03 to 0.08) | 0.01 (0.01 to 0.03) | -0.73 (-0.85 to -0.51) |
|  | Deaths | 0.1 (0.07 to 0.16) | 0.06 (0.03 to 0.12) | -0.43 (-0.68 to 0.05) | 0 (0 to 0) | 0 (0 to 0) | -0.72 (-0.84 to -0.47) |
| Malawi | Prevalence | 112.93 (87.09 to 144.71) | 228.3 (174.7 to 290.98) | 1.02 (0.93 to 1.13) | 1.33 (1.05 to 1.66) | 1.28 (0.99 to 1.59) | -0.04 (-0.07 to 0) |
|  | Incidence | 36.72 (30.16 to 45.09) | 64.82 (52.26 to 79.55) | 0.77 (0.67 to 0.85) | 0.54 (0.43 to 0.69) | 0.5 (0.39 to 0.62) | -0.08 (-0.12 to -0.05) |
|  | DALYs | 28.31 (20.16 to 39.29) | 50.45 (32.73 to 73.11) | 0.78 (0.57 to 0.96) | 0.32 (0.23 to 0.44) | 0.29 (0.19 to 0.4) | -0.1 (-0.16 to -0.04) |
|  | YLDs | 24.02 (15.72 to 35.02) | 48.57 (31.19 to 71.02) | 1.02 (0.93 to 1.13) | 0.28 (0.19 to 0.4) | 0.27 (0.18 to 0.38) | -0.04 (-0.07 to 0) |
|  | YLLs | 4.29 (2.51 to 6.85) | 1.88 (0.76 to 4.25) | -0.56 (-0.83 to 0.12) | 0.04 (0.02 to 0.06) | 0.01 (0.01 to 0.03) | -0.62 (-0.82 to -0.19) |
|  | Deaths | 0.07 (0.04 to 0.1) | 0.04 (0.02 to 0.09) | -0.42 (-0.74 to 0.27) | 0 (0 to 0) | 0 (0 to 0) | -0.57 (-0.77 to -0.03) |
| Mozambique | Prevalence | 160.07 (126.03 to 202.62) | 382.32 (292.04 to 483.17) | 1.39 (1.27 to 1.52) | 1.37 (1.09 to 1.7) | 1.39 (1.09 to 1.72) | 0.01 (-0.03 to 0.05) |
|  | Incidence | 57.59 (47.51 to 70.1) | 109.81 (90.73 to 132.84) | 0.91 (0.81 to 0.99) | 0.6 (0.47 to 0.75) | 0.53 (0.42 to 0.65) | -0.12 (-0.16 to -0.09) |
|  | DALYs | 39.17 (27.81 to 55.22) | 84.67 (54.95 to 122.75) | 1.16 (0.92 to 1.34) | 0.32 (0.23 to 0.45) | 0.31 (0.21 to 0.44) | -0.04 (-0.11 to 0.02) |
|  | YLDs | 34.05 (22.25 to 49.23) | 81.32 (52.21 to 120.03) | 1.39 (1.27 to 1.52) | 0.29 (0.2 to 0.41) | 0.3 (0.2 to 0.42) | 0.01 (-0.03 to 0.05) |
|  | YLLs | 5.13 (3.09 to 7.84) | 3.35 (1.29 to 9.25) | -0.35 (-0.72 to 0.57) | 0.03 (0.02 to 0.05) | 0.01 (0.01 to 0.04) | -0.57 (-0.8 to 0.05) |
|  | Deaths | 0.08 (0.05 to 0.14) | 0.06 (0.02 to 0.2) | -0.23 (-0.64 to 0.85) | 0 (0 to 0) | 0 (0 to 0) | -0.53 (-0.76 to 0.18) |
| Rwanda | Prevalence | 68.11 (51.58 to 87.57) | 134.59 (102.29 to 173.28) | 0.98 (0.84 to 1.08) | 1.08 (0.84 to 1.35) | 1.08 (0.84 to 1.37) | 0 (-0.04 to 0.03) |
|  | Incidence | 22.05 (18.03 to 26.71) | 40.1 (32.36 to 50.02) | 0.82 (0.69 to 0.93) | 0.45 (0.36 to 0.56) | 0.41 (0.33 to 0.51) | -0.09 (-0.12 to -0.06) |
|  | DALYs | 17.54 (12.26 to 24.64) | 29.71 (19.68 to 42.71) | 0.69 (0.41 to 0.9) | 0.27 (0.19 to 0.37) | 0.24 (0.16 to 0.34) | -0.11 (-0.2 to -0.03) |
|  | YLDs | 14.49 (9.48 to 21.23) | 28.64 (18.58 to 41.81) | 0.98 (0.84 to 1.08) | 0.23 (0.15 to 0.33) | 0.23 (0.15 to 0.33) | 0 (-0.04 to 0.03) |
|  | YLLs | 3.05 (1.55 to 5.71) | 1.07 (0.56 to 1.96) | -0.65 (-0.82 to -0.15) | 0.04 (0.02 to 0.06) | 0.01 (0.01 to 0.02) | -0.73 (-0.85 to -0.46) |
|  | Deaths | 0.05 (0.03 to 0.08) | 0.03 (0.01 to 0.05) | -0.5 (-0.74 to 0.06) | 0 (0 to 0) | 0 (0 to 0) | -0.69 (-0.83 to -0.43) |
| Somalia | Prevalence | 70.24 (52.92 to 90.2) | 179.83 (133.52 to 233.94) | 1.56 (1.42 to 1.71) | 1.02 (0.8 to 1.28) | 0.95 (0.74 to 1.19) | -0.07 (-0.11 to -0.04) |
|  | Incidence | 28.71 (23.44 to 35.39) | 72.54 (59.05 to 89.67) | 1.53 (1.41 to 1.63) | 0.55 (0.44 to 0.7) | 0.53 (0.41 to 0.66) | -0.05 (-0.09 to -0.02) |
|  | DALYs | 15.76 (10.29 to 22.65) | 38.55 (24.68 to 56.04) | 1.45 (1.29 to 1.59) | 0.23 (0.16 to 0.32) | 0.2 (0.14 to 0.29) | -0.11 (-0.16 to -0.06) |
|  | YLDs | 14.94 (9.41 to 21.53) | 38.25 (24.57 to 55.78) | 1.56 (1.42 to 1.71) | 0.22 (0.14 to 0.31) | 0.2 (0.13 to 0.29) | -0.07 (-0.11 to -0.04) |
|  | YLLs | 0.82 (0.47 to 1.42) | 0.3 (0.09 to 0.81) | -0.63 (-0.87 to -0.24) | 0.01 (0.01 to 0.02) | 0 (0 to 0.01) | -0.79 (-0.91 to -0.64) |
|  | Deaths | 0.01 (0.01 to 0.03) | 0.01 (0 to 0.02) | -0.54 (-0.82 to -0.14) | 0 (0 to 0) | 0 (0 to 0) | -0.78 (-0.9 to -0.63) |
| South Sudan | Prevalence | 67.07 (51.71 to 85.42) | 101.63 (76.72 to 130.55) | 0.52 (0.44 to 0.6) | 1.31 (1.02 to 1.63) | 1.2 (0.93 to 1.49) | -0.09 (-0.12 to -0.05) |
|  | Incidence | 19.21 (15.71 to 22.87) | 31.29 (25.6 to 38.39) | 0.63 (0.55 to 0.72) | 0.47 (0.37 to 0.59) | 0.46 (0.37 to 0.57) | -0.03 (-0.06 to 0.01) |
|  | DALYs | 16.13 (11.29 to 22.85) | 22.36 (14.61 to 32.78) | 0.39 (0.25 to 0.5) | 0.31 (0.22 to 0.43) | 0.26 (0.18 to 0.37) | -0.15 (-0.21 to -0.1) |
|  | YLDs | 14.27 (9.26 to 20.93) | 21.62 (14.07 to 31.85) | 0.52 (0.44 to 0.6) | 0.28 (0.19 to 0.4) | 0.25 (0.17 to 0.36) | -0.09 (-0.12 to -0.05) |
|  | YLLs | 1.86 (1.04 to 3.07) | 0.73 (0.33 to 1.97) | -0.61 (-0.82 to -0.08) | 0.03 (0.01 to 0.06) | 0.01 (0 to 0.02) | -0.72 (-0.86 to -0.34) |
|  | Deaths | 0.03 (0.02 to 0.06) | 0.01 (0.01 to 0.04) | -0.58 (-0.79 to -0.01) | 0 (0 to 0) | 0 (0 to 0) | -0.72 (-0.86 to -0.3) |
| Uganda | Prevalence | 161.7 (120.87 to 205.96) | 418.87 (314.41 to 544.83) | 1.59 (1.46 to 1.74) | 1.1 (0.85 to 1.38) | 1.1 (0.85 to 1.38) | 0 (-0.04 to 0.04) |
|  | Incidence | 52.36 (43.24 to 62.51) | 113.2 (93.45 to 135.32) | 1.16 (1.06 to 1.26) | 0.45 (0.36 to 0.56) | 0.39 (0.31 to 0.49) | -0.12 (-0.15 to -0.09) |
|  | DALYs | 39.3 (26.83 to 56.41) | 94.94 (62.46 to 139.01) | 1.42 (1.19 to 1.63) | 0.26 (0.18 to 0.36) | 0.25 (0.17 to 0.36) | -0.03 (-0.11 to 0.07) |
|  | YLDs | 34.4 (22.42 to 51.16) | 89.13 (57.19 to 132.69) | 1.59 (1.46 to 1.74) | 0.23 (0.16 to 0.33) | 0.23 (0.15 to 0.34) | 0 (-0.04 to 0.04) |
|  | YLLs | 4.9 (3.02 to 7.98) | 5.81 (2.56 to 16.99) | 0.19 (-0.46 to 1.77) | 0.02 (0.01 to 0.05) | 0.02 (0.01 to 0.06) | -0.29 (-0.62 to 0.97) |
|  | Deaths | 0.08 (0.04 to 0.16) | 0.11 (0.05 to 0.35) | 0.4 (-0.28 to 2.64) | 0 (0 to 0) | 0 (0 to 0) | -0.27 (-0.66 to 1.3) |
| United Republic of Tanzania | Prevalence | 283.23 (220.59 to 356.74) | 666.98 (511.68 to 850.16) | 1.35 (1.23 to 1.47) | 1.27 (0.99 to 1.57) | 1.26 (0.98 to 1.57) | -0.01 (-0.05 to 0.03) |
|  | Incidence | 82.79 (67.76 to 99.87) | 174.9 (142.11 to 211.38) | 1.11 (1.02 to 1.2) | 0.46 (0.37 to 0.58) | 0.42 (0.33 to 0.52) | -0.1 (-0.13 to -0.07) |
|  | DALYs | 75.3 (54.83 to 105.52) | 150.33 (98.03 to 218.94) | 1 (0.73 to 1.23) | 0.32 (0.23 to 0.43) | 0.29 (0.19 to 0.41) | -0.1 (-0.19 to -0.03) |
|  | YLDs | 60.25 (39.72 to 89.14) | 141.89 (91.59 to 208.97) | 1.35 (1.23 to 1.47) | 0.27 (0.18 to 0.39) | 0.27 (0.18 to 0.38) | -0.01 (-0.05 to 0.03) |
|  | YLLs | 15.05 (8.28 to 23.09) | 8.44 (4.26 to 17.32) | -0.44 (-0.73 to 0.24) | 0.05 (0.03 to 0.07) | 0.02 (0.01 to 0.04) | -0.63 (-0.81 to -0.29) |
|  | Deaths | 0.24 (0.15 to 0.36) | 0.17 (0.08 to 0.37) | -0.29 (-0.63 to 0.46) | 0 (0 to 0) | 0 (0 to 0) | -0.6 (-0.8 to -0.25) |
| Zambia | Prevalence | 95.97 (74.06 to 122.12) | 244.76 (186.78 to 313.31) | 1.55 (1.43 to 1.66) | 1.4 (1.1 to 1.74) | 1.37 (1.08 to 1.7) | -0.02 (-0.05 to 0.02) |
|  | Incidence | 27.38 (22.57 to 33.07) | 61.76 (50.5 to 75.27) | 1.26 (1.14 to 1.36) | 0.52 (0.41 to 0.65) | 0.46 (0.37 to 0.57) | -0.11 (-0.14 to -0.08) |
|  | DALYs | 26.48 (18.69 to 36.66) | 55.73 (37.38 to 80.82) | 1.1 (0.77 to 1.4) | 0.36 (0.26 to 0.49) | 0.32 (0.22 to 0.44) | -0.12 (-0.2 to -0.04) |
|  | YLDs | 20.42 (13.26 to 30.09) | 52.08 (33.76 to 76.21) | 1.55 (1.43 to 1.66) | 0.3 (0.2 to 0.42) | 0.29 (0.19 to 0.42) | -0.02 (-0.05 to 0.02) |
|  | YLLs | 6.07 (2.9 to 9.74) | 3.65 (2.17 to 6.94) | -0.4 (-0.71 to 0.34) | 0.06 (0.04 to 0.09) | 0.03 (0.02 to 0.05) | -0.6 (-0.78 to -0.31) |
|  | Deaths | 0.09 (0.06 to 0.14) | 0.07 (0.04 to 0.14) | -0.2 (-0.56 to 0.5) | 0 (0 to 0) | 0 (0 to 0) | -0.56 (-0.74 to -0.23) |
| Southern Sub-Sahara |  |  |  |  |  |  |  |
| Botswana | Prevalence | 20.31 (15.69 to 25.77) | 39.71 (31.15 to 49.69) | 0.96 (0.82 to 1.1) | 1.64 (1.3 to 2.03) | 1.66 (1.32 to 2.04) | 0.08 (0 to 0.16) |
|  | Incidence | 4.2 (3.48 to 5.03) | 7.98 (6.34 to 9.94) | 0.9 (0.74 to 1.05) | 0.44 (0.35 to 0.53) | 0.39 (0.32 to 0.47) | 0.05 (-0.01 to 0.13) |
|  | DALYs | 9.94 (6.66 to 14.35) | 15.83 (9.37 to 26.85) | 0.59 (-0.07 to 1.87) | 0.83 (0.52 to 1.2) | 0.63 (0.4 to 1.02) | 0.05 (-0.01 to 0.11) |
|  | YLDs | 4.32 (2.86 to 6.37) | 8.45 (5.62 to 12.28) | 0.96 (0.82 to 1.1) | 0.35 (0.23 to 0.5) | 0.35 (0.24 to 0.5) | 0.04 (-0.02 to 0.1) |
|  | YLLs | 5.62 (2.57 to 10.05) | 7.38 (2.36 to 17.81) | 0.31 (-0.59 to 2.74) | 0.48 (0.19 to 0.85) | 0.28 (0.1 to 0.65) | -0.02 (-0.06 to 0.03) |
|  | Deaths | 0.1 (0.04 to 0.18) | 0.13 (0.04 to 0.32) | 0.24 (-0.63 to 2.7) | 0.01 (0 to 0.02) | 0.01 (0 to 0.01) | -0.02 (-0.07 to 0.03) |
| Eswatini | Prevalence | 12.5 (9.62 to 15.55) | 18.83 (14.64 to 23.59) | 0.51 (0.42 to 0.61) | 1.67 (1.33 to 2.05) | 1.65 (1.32 to 2.02) | 0.05 (-0.04 to 0.13) |
|  | Incidence | 2.79 (2.3 to 3.35) | 3.91 (3.19 to 4.83) | 0.4 (0.32 to 0.5) | 0.48 (0.39 to 0.6) | 0.44 (0.36 to 0.53) | -0.02 (-0.08 to 0.05) |
|  | DALYs | 6.14 (4.3 to 9) | 9.37 (5.42 to 19.43) | 0.52 (-0.14 to 2.07) | 0.83 (0.54 to 1.25) | 0.79 (0.47 to 1.58) | -0.12 (-0.17 to -0.08) |
|  | YLDs | 2.66 (1.77 to 3.82) | 4.01 (2.65 to 5.86) | 0.51 (0.42 to 0.61) | 0.35 (0.24 to 0.5) | 0.35 (0.23 to 0.5) | -0.02 (-0.08 to 0.03) |
|  | YLLs | 3.48 (2.04 to 6.27) | 5.36 (1.73 to 15.22) | 0.54 (-0.51 to 3.5) | 0.47 (0.22 to 0.9) | 0.44 (0.15 to 1.27) | 0 (-0.05 to 0.05) |
|  | Deaths | 0.06 (0.03 to 0.11) | 0.09 (0.03 to 0.27) | 0.61 (-0.49 to 3.91) | 0.01 (0 to 0.02) | 0.01 (0 to 0.02) | 0.03 (-0.02 to 0.08) |
| Lesotho | Prevalence | 23.65 (18.82 to 29.39) | 30.44 (23.9 to 38.22) | 0.29 (0.22 to 0.36) | 1.64 (1.31 to 2.01) | 1.62 (1.28 to 2) | -0.12 (-0.49 to 0.58) |
|  | Incidence | 6.58 (5.45 to 7.96) | 7.06 (5.75 to 8.64) | 0.07 (0.01 to 0.13) | 0.54 (0.44 to 0.67) | 0.47 (0.39 to 0.58) | 0.06 (-0.4 to 1.14) |
|  | DALYs | 7.61 (5.5 to 10.74) | 9.19 (6.1 to 13.47) | 0.21 (-0.11 to 0.56) | 0.53 (0.38 to 0.74) | 0.49 (0.33 to 0.71) | -0.01 (-0.27 to 0.28) |
|  | YLDs | 5.03 (3.37 to 7.26) | 6.48 (4.25 to 9.52) | 0.29 (0.22 to 0.36) | 0.35 (0.23 to 0.49) | 0.35 (0.23 to 0.5) | -0.13 (-0.45 to 0.43) |
|  | YLLs | 2.58 (1.43 to 4.73) | 2.71 (0.95 to 5.62) | 0.05 (-0.6 to 1.22) | 0.18 (0.08 to 0.34) | 0.14 (0.05 to 0.29) | -0.23 (-0.53 to 0.23) |
|  | Deaths | 0.05 (0.02 to 0.1) | 0.05 (0.02 to 0.1) | -0.05 (-0.63 to 1.24) | 0 (0 to 0.01) | 0 (0 to 0.01) | 0.23 (-0.19 to 0.85) |
| Namibia | Prevalence | 21.97 (17 to 27.64) | 39.48 (30.69 to 49.81) | 0.8 (0.7 to 0.9) | 1.67 (1.31 to 2.05) | 1.65 (1.31 to 2.03) | 0.08 (0 to 0.16) |
|  | Incidence | 4.54 (3.75 to 5.46) | 7.7 (6.24 to 9.42) | 0.7 (0.59 to 0.79) | 0.43 (0.35 to 0.54) | 0.39 (0.32 to 0.48) | 0.05 (-0.01 to 0.13) |
|  | DALYs | 10.06 (7.08 to 13.85) | 15.11 (9.35 to 25.91) | 0.5 (-0.05 to 1.48) | 0.76 (0.51 to 1.04) | 0.62 (0.39 to 1.06) | 0.05 (-0.01 to 0.11) |
|  | YLDs | 4.67 (3.12 to 6.74) | 8.4 (5.56 to 12.08) | 0.8 (0.7 to 0.9) | 0.35 (0.24 to 0.5) | 0.35 (0.23 to 0.49) | 0.04 (-0.02 to 0.1) |
|  | YLLs | 5.39 (2.89 to 8.65) | 6.71 (2.14 to 16.72) | 0.25 (-0.62 to 2.28) | 0.4 (0.19 to 0.66) | 0.27 (0.09 to 0.65) | -0.02 (-0.06 to 0.03) |
|  | Deaths | 0.1 (0.05 to 0.16) | 0.12 (0.04 to 0.3) | 0.21 (-0.62 to 2.15) | 0.01 (0 to 0.02) | 0.01 (0 to 0.01) | -0.02 (-0.07 to 0.03) |
| South Africa | Prevalence | 680.79 (521.46 to 857.24) | 1027.96 (789.98 to 1301.05) | 0.51 (0.44 to 0.58) | 1.87 (1.46 to 2.32) | 1.78 (1.39 to 2.23) | -0.28 (-0.77 to 1.06) |
|  | Incidence | 138.34 (113.38 to 169.73) | 212.8 (169.51 to 269.9) | 0.54 (0.45 to 0.62) | 0.45 (0.37 to 0.57) | 0.4 (0.32 to 0.5) | 0.07 (-0.66 to 2.14) |
|  | DALYs | 318.93 (181.5 to 565.02) | 377.23 (211.52 to 571.64) | 0.18 (-0.27 to 0.89) | 0.91 (0.52 to 1.58) | 0.62 (0.36 to 0.92) | -0.14 (-0.67 to 0.82) |
|  | YLDs | 144.82 (92.87 to 209.44) | 218.68 (143.17 to 314.21) | 0.51 (0.44 to 0.58) | 0.4 (0.26 to 0.56) | 0.38 (0.25 to 0.54) | -0.28 (-0.78 to 0.9) |
|  | YLLs | 174.12 (46.42 to 427.14) | 158.55 (32.66 to 353.97) | -0.09 (-0.73 to 1.32) | 0.51 (0.13 to 1.19) | 0.24 (0.06 to 0.52) | -0.41 (-0.83 to 0.51) |
|  | Deaths | 3.52 (0.87 to 7.77) | 3.04 (0.69 to 6.53) | -0.14 (-0.71 to 1.5) | 0.01 (0 to 0.02) | 0.01 (0 to 0.01) | 0.42 (-0.28 to 1.73) |
| Zimbabwe | Prevalence | 143.37 (110.32 to 181.57) | 211.66 (165.26 to 268.8) | 0.48 (0.4 to 0.55) | 1.48 (1.17 to 1.82) | 1.41 (1.13 to 1.74) | -0.31 (-0.8 to 1.04) |
|  | Incidence | 32.29 (26.92 to 38.69) | 50.18 (41.85 to 59.6) | 0.55 (0.48 to 0.63) | 0.43 (0.36 to 0.52) | 0.43 (0.36 to 0.5) | 0.12 (-0.64 to 2.43) |
|  | DALYs | 70.42 (45.75 to 93.18) | 130.4 (93.03 to 192.03) | 0.85 (0.22 to 1.79) | 0.9 (0.51 to 1.22) | 1.02 (0.7 to 1.5) | -0.22 (-0.7 to 0.83) |
|  | YLDs | 30.5 (20.33 to 44.08) | 45.02 (29.84 to 65.39) | 0.48 (0.4 to 0.55) | 0.31 (0.21 to 0.44) | 0.3 (0.2 to 0.43) | -0.3 (-0.78 to 0.82) |
|  | YLLs | 39.93 (17.16 to 59.62) | 85.37 (50.79 to 140.96) | 1.14 (0.09 to 3.12) | 0.58 (0.2 to 0.89) | 0.72 (0.4 to 1.19) | -0.44 (-0.81 to 0.63) |
|  | Deaths | 1.05 (0.35 to 1.62) | 2.01 (1.11 to 3.28) | 0.91 (0.01 to 3.09) | 0.02 (0.01 to 0.03) | 0.02 (0.01 to 0.04) | 0.27 (-0.33 to 1.71) |
